# Diselenophosphate Ligands as a Surface Engineering Tool in PdH-Doped Silver Superatomic Nanoclusters

**DOI:** 10.1021/acs.inorgchem.3c04253

**Published:** 2024-01-22

**Authors:** Yu-Rong Ni, Michael N. Pillay, Tzu-Hao Chiu, Jagadeesh Rajaram, Ying-Yann Wu, Samia Kahlal, Jean-Yves Saillard, C. W. Liu

**Affiliations:** † Department of Chemistry, National Dong Hwa University, Hualien 97401 Taiwan, Republic of China; ‡ Univ Rennes CNRS, ISC-UMR 6226, F-35000 Rennes, France

## Abstract

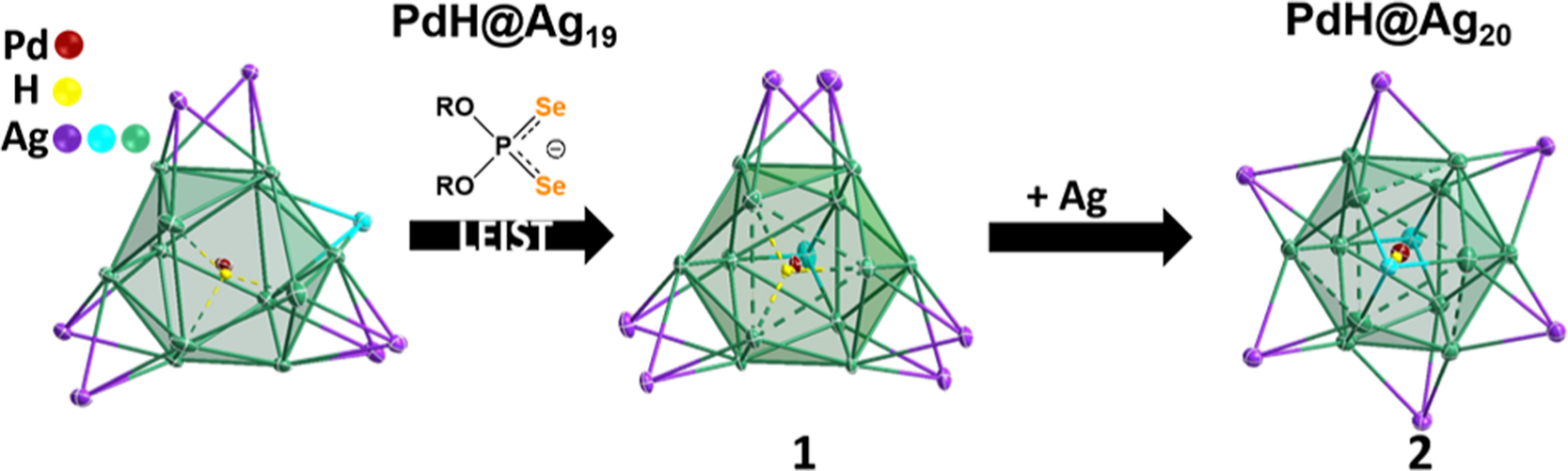

The first hydride-doped Pd/Ag superatoms stabilized by selenolates are reported: [PdHAg_19_(dsep)_12_] [dsep = Se_2_P(O^*i*^Pr)_2_] **1** and [PdHAg_20_(dsep)_12_]^+^**2**. **1** was derived from the targeted transformation of [PdHAg_19_(dtp)_12_] [dtp = S_2_P(O^*i*^Pr)_2_] by ligand exchange, whereas **2** was obtained from the addition of trifluoroacetic acid to **1**, resulting in a symmetric redistribution of the capping silver atoms. The transformations are all achieved while retaining an 8-electron superatomic configuration. VT-NMR attests to the good stability of the NCs in solution, and single-crystal X-ray diffraction reveals the crucial role that the interstitial hydride plays in directing the position of the capping silver atoms. The total structures are reported alongside their electronic and optical properties. **1** and **2** are phosphorescent with a lifetime of 73 and 84 μs at 77 K, respectively. The first antibacterial activity data for superatomic bimetallic Pd/Ag nanoclusters are also reported.

## Abstract 2

Two novel PdH-doped silver superatoms, {PdHAg_19_[Se_2_P(O^*i*^Pr)_2_]_12_} **1** and [PdHAg_20_{Se_2_P(O^*i*^Pr)_2_}_12_] **2**, are reported. Derived from the ligand exchange transformation of {PdHAg_19_[S_2_P(O^*i*^Pr)_2_]_12_}, a stable 8-electron superatomic configuration is maintained. The interstitial hydride plays a crucial role in directing the metal framework and results in the isolation of a highly symmetric arrangement. VT-NMR indicates that the solid-state configuration is maintained in solution. The electronic structure is investigated computationally, with optical and antibacterial properties reported.

## Introduction

Crucial to the application of atomically precise nanoclusters (NCs) is the ability to controllably modify their structural features and, in turn, their physicochemical properties. The search for new configurations is motivated by the diversity of applications, which, inter alia, includes vital renewable energy technologies^1,2^ and catalysis.^3−7^ Although creating novel architectures is always essential, synthetic protocols that provide NCs with incremental atomically precise changes (in nuclearity, symmetry, and surface nature) are of significant importance and often a more challenging aspect of NC chemistry. Therefore, it is paramount to understand and control these manipulations from a fundamental point of view and unlock the potential of NCs in future technologies.

Coinage metals are of particular interest in the formation of ligand-protected NCs, notably because of their rich optical properties.^8^ Since our first report on silver superatomic icosahedral frameworks,^9^ our priority has been to develop synthetic protocols for the optimal and precise control of the NC architecture and composition.^9−11^ Ligand exchange-induced structure transformation (LEIST) has been an efficient tool in fine-tuning NC morphology and has been the subject of a review.^12^ We have previously established the ability of diselenophosphate (dsep) to completely substitute dithiophosphonates (dtp) in silver and copper frameworks^13^ and have expanded the methodology to group 10-doped NCs.^14^ First, we isolated a Pd-doped NC of the type {Pd@Ag_20_[Se_2_P(O^*i*^Pr)_2_]_12_}, demonstrating the viability of LEIST surface modification in these systems.^14^ This was followed by the isolation of a PtH derivative {(PtH)@Ag_19_[Se_2_P(O^*i*^Pr)_2_]_12_} (structure confirmed by neutron diffraction), demonstrating a remarkable configurational change of the metallic skeleton.^15^ Entrapping a PdH motif in superatomic coinage metal constructs has only been realized recently. It is exceedingly rare for silver-rich constructs^16−18^ and so far unknown in the absence of dsep-stabilizing ligands. Inserting PdH units into a Ag_12_ icosahedron, itself surrounded by an outer capping layer, has been a focus of our current research.^19^ The stability of these closed-shell-doped systems can be rationalized within the *superatom* model, with the dopant contributing to the stability of these assemblies.^15,19^ We report below the transformation of PdH-doped metallic frameworks with LEIST to yield unique structural conformations that would be otherwise inaccessible. Hydrides are well-known for their invaluable electron-donor ability, thus reducing metal atoms to give rise to electron-precise mixed-valent *superatoms*. They can also behave as a dopant within the *superatom* core, thus contributing one electron to the *superatom* count,^10,11,13,15,20−23^ whereas, in these systems, the Pd atom contribution to the *superatomic* count is zero.^24−27^ In this report, we provide experimental evidence for the ability of the hydride to dictate the configuration of the outer metal/ligand shell.

Two novel luminescent 8-electron superatoms: [PdHAg_19_(dsep)_12_] **1** and [PdHAg_20_(dsep)_12_](TFA) ([**2**](TFA); TFA = trifluoroacetate), are isolated and characterized by single-crystal X-ray diffraction (SCXRD), X-ray photoelectron spectroscopy (XPS), ESI-TOF-MS, UV–vis, and NMR spectroscopy.

## Results and Discussion

**Scheme 1:**
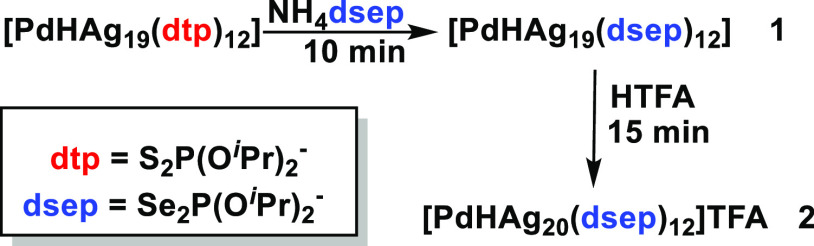
Ligand Exchange Protocol for the Surface Modification of Pd-Doped NC and the Controlled Addition of a Single Ag Atom

The reaction of the dsep ligand with [PdHAg_19_(dtp)_12_] in THF results in the formation of **1**, as shown in Scheme 1. The reaction is light-sensitive and must be performed in a reasonably dark environment. The conversion is not quantitative, and purification by column chromatography yielded **1** in 54%. It should be noted that the direct co-reduction method in the presence of dsep as the ligand does not yield the target product. Once isolated, **1** shows moderate stability and is amenable to transformations similar to those we reported for the dtp analogues.^19^ Briefly, the formation of **2** is induced by adding trifluoroacetic acid (HTFA), which promotes the intercluster transfer of silver atoms. An 8-electron count is maintained throughout the conversion, and **2** is obtained in 62% yield.

**Figure 1:**
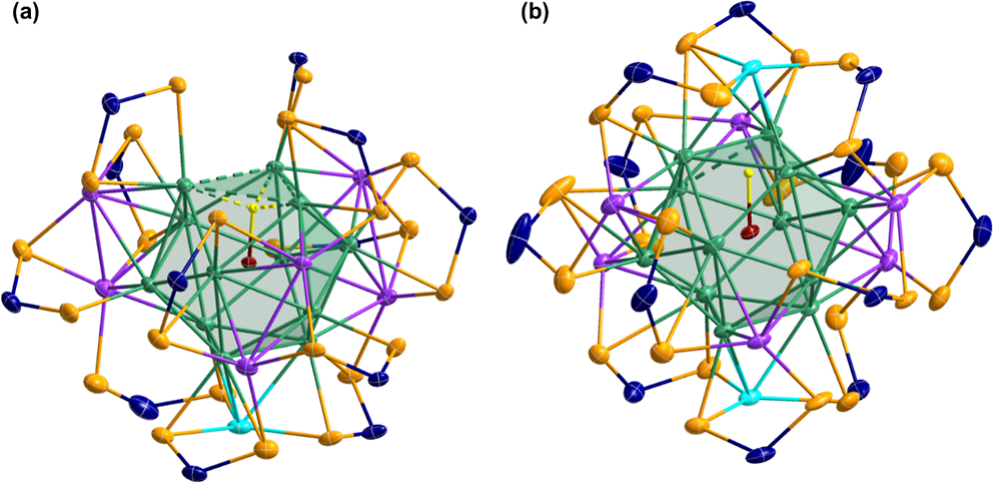
Molecular representation of **1** (a) and **2** (b) (atom color code: blue, P; orange, Se; purple, capping Ag (Ag_cap_); pale blue, capping Ag along the *C*_3_ axis (Ag_cap_′); green, icosahedron Ag; brown, Pd; and yellow, H.).

SCXRD data collected for **1** and **2** were of sufficient quality that the position of the hydride could be assigned from the residual electron densities. Moreover, their positions were supported by density functional theory (DFT) calculations (vide infra). Selected bond lengths are given in Table 1, with molecular representations illustrated in Figures 1, S7 and S8. Refinement and crystallographic data are listed in Table S1. A common feature in both structures is a 12-atom silver icosahedron centered by a PdH unit. The encapsulated PdH motif distorts the Ag_12_ icosahedron, highlighted by continuous symmetry measure (CSM) values of 0.12 and 0.09 for **1** and **2**, respectively. The distortion is lower in **2** compared to that in **1** and significantly lower than in the dtp analogue (**2dtp**), correlating to the increase in symmetry observed in solution NMR. Furthermore, an assessment of the Ag–Ag bond lengths given in Figure S5 emphasizes the significant distortion observed in **1**, with Ag···Ag contacts extending to 3.3 Å. The driving force behind the transformation of the metallic framework is related to the geometrical differences between the S–P–S and Se–P–Se moieties. The size difference between Se and S plays a crucial role in the ligand bite distance.

**Table 1:** Selected Bond Lengths and Geometrical Parameters for **1** and **2**

	**1**	**2**
CSM	0.12	0.09
Pd_cen_–Ag_ico_, Å	2.7589(8)–2.8895(8)	2.691(3)–2.876(3)
	avg. 2.8042(8)	avg. 2.788(3)
Ag_ico_–Ag_ico_, Å	2.8427(9)–3.3021(9)	2.854(3)–3.002(4)
	avg. 2.9490(8)	avg. 2.9312(3)
Ag_ico_–Ag_cap_, Å	2.8931(9)–3.0549(8)	2.880(3)–3.042(3)
	avg. 2.9697(9)	avg. 2.964(3)
Ag_ico_–Ag_cap_′, Å	2.9471(8)–2.9765(10)	2.917(3)–3.008(2)
	avg. 2.9630(8)	avg. 2.963(3)

**Figure 2:**
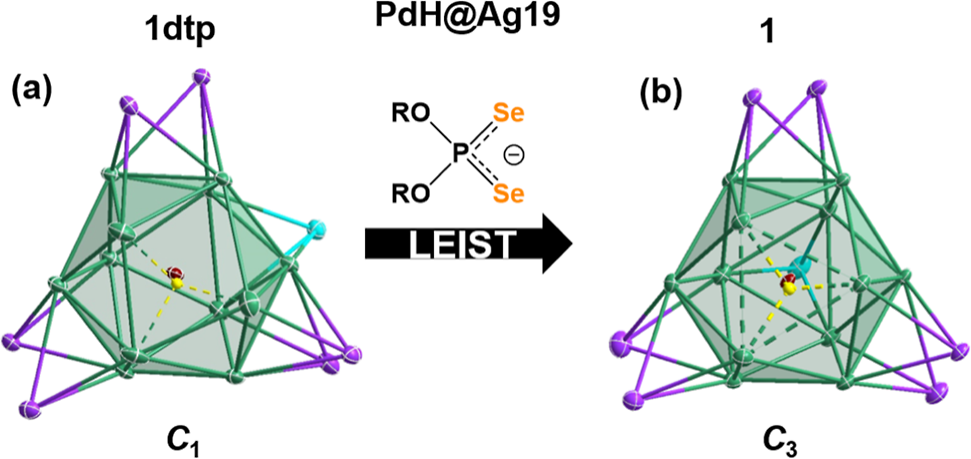
Metallic framework presented in parent cluster [PdHAg_19_(dtp)_12_] (a) and the improved symmetric assembly presented in **1** (b) after LEIST.

Considering the separations between the two chalcogens, the Se···Se distances are ca. 3.66 Å, while the S···S distance is ca. 3.39 Å. The seemingly trivial 0.27 Å difference plays a crucial role in allowing a more symmetrical arrangement of the capping atoms in the solid state. Also, this explains the lack of isomerization in the solution. Unlike the dtp analogues, which can only achieve these symmetric arrangements in solution, the dsep analogues stabilize *C*_3_ configurations in the solid state, Figure 2.

**Figure 3:**
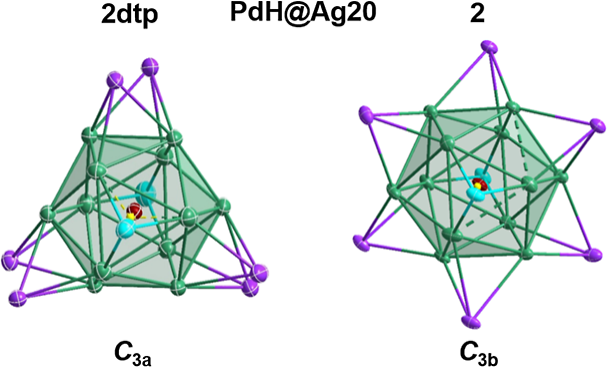
Different metallic frameworks of **2dtp** (*C*_3a_) and **2** (*C*_3b_), illustrating the transformation driven by the ligand nature and the encapsulated hydride.

Also of note is the effect of the kernel composition on the positions of the capping atoms. The inclusion of the interstitial hydride not only plays a crucial role in NC stabilization but also has a significant structural effect on the metallic shell arrangement. Comparing Ag_20_ shells in Pd@Ag_20_(dtp_12_),^28^ Pd@Ag_20_(dsep)_12_,^14^ PdH@Ag_20_(dtp)_12_,^19^ and PdH@Ag_20_(dsep)_12_ (**2**), we found the hydride to be directive toward a particular Ag_20_ framework. In the absence of a hydride dopant, crystal growth of Pd@Ag_20_(dsep)_12_ results in a cocrystal consisting of two distinct *C*_3a_ and *C*_3b_ metallic frameworks, Figure 3.^14^

In the case of **2**, the *C*_3b_ arrangement is exclusively obtained, while the *C*_3a_ structure is obtained in the case of PdH@Ag_20_(dtp)_12_.^19^ The Pd–H bond axis dictates the orientation of the *C*_3_ rotational axis, thus affording fewer possible positions to occupy for the remaining six capping atoms while retaining a symmetric arrangement.

Combining this effect with the limited reach of the dtp ligand, the occupation of adjacent triangular faces is necessary to passivate the assembly. In **2**, the dsep ligands allow the capping atoms to occupy faces further away, resulting in the *C*_3b_ structure. Furthermore, the arrangement of the capping atoms in **2** creates several equivalent interstitial sites for occupation by the hydride, which relates to the dynamic behavior observed in variable-temperature (VT)-NMR. The change in the configuration and the surface of the cluster has a profound effect on the physical properties of the NC, resulting in both NCs being emissive at 77 K.

**Figure 4:**
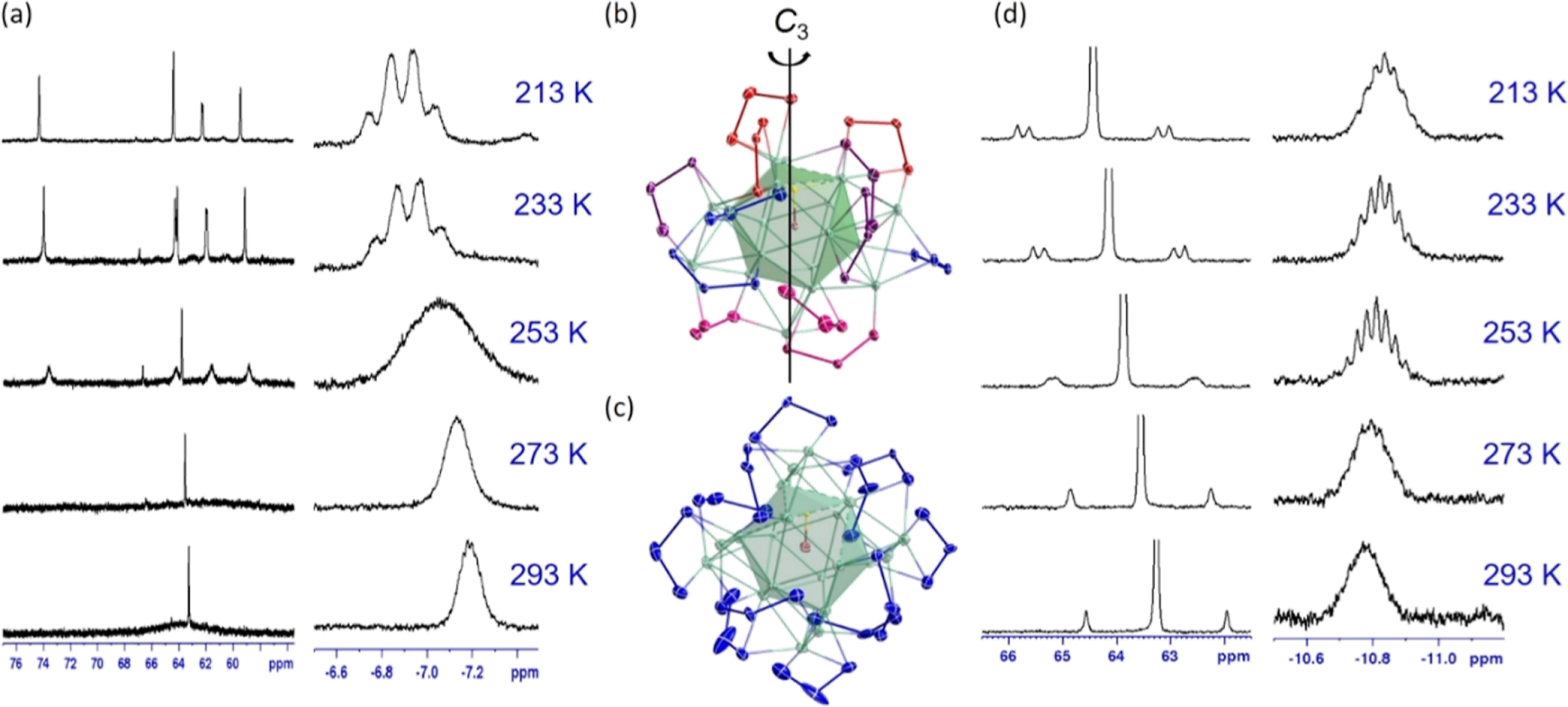
(a) ^31^P{^1^H} NMR (CD_2_Cl_2_) (left) of **1** indicating the lability of the ligand environment at 293 K and ^1^H NMR (right) with quartet resonance observed at 213 K. Color-coded ligand environments corresponding to the four phosphorus resonances observed in **1** (b) and the single ligand environment observed in **2** (c). (d) ^31^P{^1^H} NMR (CD_2_Cl_2_) (left) indicating the pseudo-symmetrically equivalent ligand environment maintained over the range of 293–213 K and the dynamic hydride environment observed in the ^1^H NMR (right) due to the movement of the hydride within the Ag_12_ icosahedron.

In our investigations of the isomeric distributions in the thiolate analogues, we found the solid state confined to one single configuration, whereas a complex isomer mixture was identified in solution from low-temperature NMR experiments.^19^ In stark contrast to their dtp analogues, the dsep species are substantially more rigid in solution at low temperatures. In the present series, no competing isomerization was identified in solution up to 213 K. In both cases, the dsep ligand induces the formation of the more symmetrical isomer, which is substantially more rigid in solution, as is evident in the VT-NMR, shown in Figure 4.

The low-temperature ^31^P NMR spectrum of **1** shows four nonequivalent resonances (Figure 4a), which correspond directly to its solid-state structure of *C*_3_ symmetry, wherein four distinct phosphorus environments are present, Figure 4b. In **2**, a single ^31^P resonance is observed during the VT-NMR (Figure 4d), indicating that the 12 dsep ligands are engaged in the same coordination mode (μ_3_: η_2_, η_1_), Figure 4c. The pair of selenium satellite peaks at 213 K corresponds to the highly symmetrical configuration of **2**, with two chemically and magnetically inequivalent Se atoms bonded to the same P atom, Figure 4d. The ^31^P NMR is significantly downfield compared to dtp analogues, with resonances observed at 63.63 (^1^*J*_PSe_ = 633.36 Hz) and 63.26 (^1^*J*_PSe_ = 635.44 Hz) for **1** and **2**, respectively, Figure S4a,b. The integral values of the ^1^H NMR signals in both **1** and **2** correlate to a ratio of 12 dsep ligands to a single hydride, Figure S2. Hydride resonances for **1** and **2** are significantly different, −7.2 and −10.7 ppm, respectively. The 3.5 ppm disparity in the hydride signal further emphasizes the susceptibility of the interstitial hydride environment to the nature of the external silver shell. Thus, not only do the hydride resonances confirm their presence, but they also provide a diagnostic tool for differentiating metallic arrangements. The hydride resonance assignments are confirmed by ^2^H NMR of their deuterated derivatives **1**_**D**_ and **2**_**D**_, which resonate at −7.1 and −10.6 ppm, respectively, in Figure S3.

At ambient conditions, the single broad hydride peak in **1** and **2** shows no definition. However, a reduction in temperature results in the formation of a quartet at −6.8 ppm for **1**. The quartet resonance has been established as resulting from the hydride coupling with three equivalent Ag atoms defining an icosahedron face while occupying an interstitial position inside a PdAg_3_ tetrahedron.^19^ In contrast, the hydride resonance in **2** does not form a quartet at low temperatures, and the peak is maintained as a multiplet.

This is due to the equivalent (symmetrically and energetically) sites for the hydride to reside. The magnetically equivalent ^31^P nuclei indicate the formation of a *T*_h_-symmetric metallic skeleton, which is, rigorously speaking, impossible because of the nonpoint PdH central unit. However, in solution and on an NMR time scale, the hydride couples with all of the triangular Ag_3_ faces of the icosahedron, indicating it is able to migrate even at low temperatures. The TFA^–^ anion in **2** was further confirmed by ^19^F NMR, with a single resonance observed at −76.5 ppm, Figure S4c.

**Figure 5:**
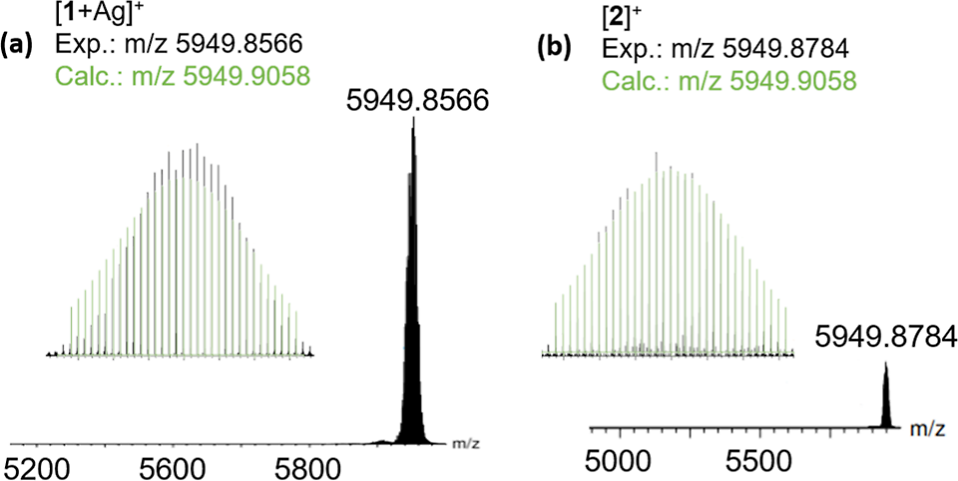
Experimental (black) and simulated (green) ESI-TOF-MS spectra for **1** (a) and **2** (b).

The compositions of clusters **1** and **2** were confirmed by ESI-TOF-MS, and a good correlation between experimental and simulated isotopic distribution patterns was observed, Figure 5. The mass spectrum of **1** shows a prominent *m*/*z* peak at 5949.8566 Da assigned to the silver adduct [**1** + Ag]^+^ (calcd 5949.9058 Da). Attempts to prepare alternative adducts were unsuccessful, with the addition of tertiary butyl ammonium or cesium cations prior to analysis not yielding the desired adducts and consistently indicating the formation of [**1** + Ag]^+^. This further emphasizes the propensity of **1** to add an additional Ag atom and the preference of the dsep ligand to bind to silver. The cationic [**2**]^+^ molecular ion peak is observed at *m*/*z* 5949.8784 Da (calcd 5949.9058 Da).

The minimal fragmentation observed in the spectra is a testament to the substantial stability of the dsep derivatives, which show remarkable resistance to fragmentation in solution, even under the harsh conditions required for ionization.

The deuterated derivatives follow a similar fragmentation pattern with adduct formation [**1**_**D**_ + Ag]^+^*m*/*z* 5950.0186 Da (calc. 5950.8991 Da) and [**2**_**D**_]^+^*m*/*z* 5950.8874 Da (calc. 5950.8991 Da), Figure S1. Importantly, the increase in the *m*/*z* ratio confirms the inclusion of a single hydride within both **1** and **2**.

### Optical Properties

**Figure 6:**
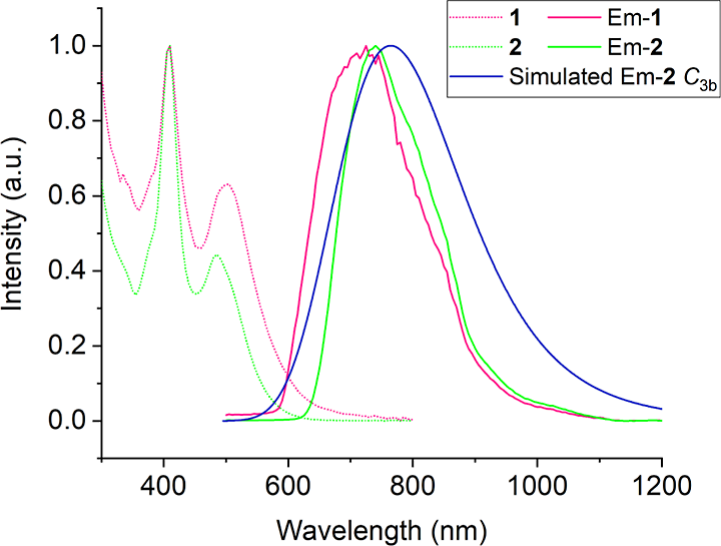
UV–vis absorption and emission spectra for **1**–**2** (λ_ex_ = 448 nm) recorded in 2-MeTHF. DFT-simulated phosphorescence spectrum of the *C*_3b_ isomer of **2**.

The contributions from the 12 dsep ligands dominate the optical properties, with both NCs having relatively similar absorption, irrespective of the modified kernel. The UV–vis spectra share a common peak centered at ca. 410 nm, with minimally different additional bands at 500 and 485 nm for **1** and **2**, respectively (Figure 6).

The second band significantly differs from dtp variants in profile, which contained a major band at 405 nm and shoulder peaks at 455 and 462 nm for **1** and **2**, respectively.^19^ Intriguing is the influence of the hydride on the emissive state, considering that the dtp analogues containing hydride-doped cores were found to be nonemissive.^19^ The additional silver atom results in a marginal red shift in the λ_em_ from 725 nm in **1** to 739 nm in **2**. The lifetime of the emission for **1** and **2** is 73 and 84 μs, respectively, Figure S6. A comparison of the emission wavelength (λ_em_) for structural-related NCs containing related dopants is presented in Table 2. In broad terms, the dsep analogues can achieve relatively blue-shifted emission wavelengths compared to their dtp counterparts.

**Table 2:** Emission Trend in Superatomically Doped Clusters

NC	dopant	ligand	λ_em_/nm	ref
{PtHAg_19_[Se_2_P(O^*i*^Pr)_2_]_12_}	PtH	dsep	636	(15)
{PdAg_20_[Se_2_P(O^*n*^Pr)_2_]_12_}	Pd	dsep	702	(14)
{PdAg_20_[Se_2_P(O^*i*^Pr)_2_]_12_}	Pd	dsep	712	(14)
{PdHAg_19_[Se_2_P(O^*i*^Pr)_2_]_12_}	PdH	dsep	725	this work
{PdHAg_20_[Se_2_P(O^*i*^Pr)_2_]_12_}^+^	PdH	dsep	739	this work
{PdAg_20_[S_2_P(O^*i*^Pr)_2_]_12_}	Pd	dtp	741	(14)
{PdAg_20_[S_2_P(O^*n*^Pr)_2_]_12_}	Pd	dtp	748	(28)
{AuAg_19_[S_2_P(O^*n*^Pr)_2_]_12_}	Au	dtp	782	(20)
{AuAg_20_[S_2_P(O^*n*^Pr)_2_]_12_}^+^	Au	dtp	745	(20)
{Ag_20_[S_2_P(O^*n*^Pr)_2_]_12_}	Ag	dtp	835	(20)
{PdAg_21_[S_2_P(O^*i*^Pr)_2_]_12_}^+^	Pd	dtp	842	(19)

This indicates the precision that can be afforded by the addition of single silver atoms to the metal framework, controllably moving the emission wavelength over a few nm.

### Computational Analysis

DFT calculations were performed on **1** and **2** at the BP86/Def2-TZVP level of calculations (see the Computational Details). For the sake of computational limits, the dsep ligands were replaced by Se_2_PH_2_, a simplification which has been proved to be reasonable in many past investigations.^10,11,13−16,19−22,28^ The optimized geometries of **1** and **2** are in good agreement with their experimental counterparts and fully confirm the hydride locations (compare Tables 2 and 3).

**Table 3:** Selected DFT-Computed Data for the Optimized *C*_3_ Geometries of **1** (*C*_3a_) and **2** (*C*_3b_) Correspond to Their SCXRD Structures^a^

	HOMO–LUMO gap (eV)	NAO charges (avg.)				distances [WBI] (avg.)					
		H	Pd	Ag_ico_	Ag_cap_	Pd–H	Ag_ico_–H	Pd_cen_–Ag_ico_	Ag_ico_–Ag_ico_	Ag_ico_–Ag_cap_	Ag_ico_–Ag_cap_′
**1**	1.63	–0.44	–0.94	+0.33	+0.66	1.710 [0.142]	1.994 [0.108]	2.887 [0.133]	3.005 [0.082]	3.104 [0.044]	3.091 [0.030]
**2**	1.78	–0.45	–0.94	+0.32	+0.67	1.706 [0.120]	1.987 [0.123]	2.887 [0.137]	3.000 [0.082]	3.082 [0.037]	3.102 [0.030]

a Average distances are in Å, and their corresponding Wiberg bond indices (WBI) are in brackets.

**Figure 7:**
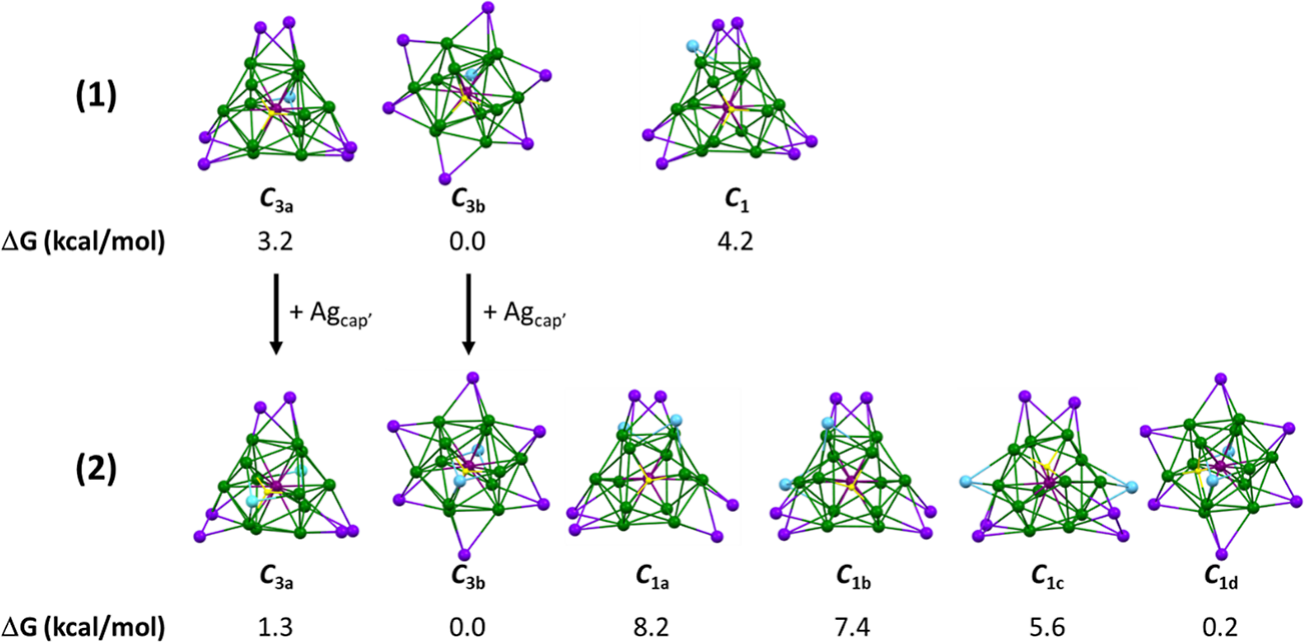
Metal frameworks of the computed isomers of **1** (top) and **2** (bottom), with their free energies at 298 K (Δ*G*). The *C*_3a,b_ and *C*_1-c_ labels refer to the definitions given in ref (19) in the main text. The vertical arrows illustrate the structure relationships between the *C*_3a_ and *C*_3b_ structures of **1** and **2** by addition on **1** of a Ag_cap_′ atom (in blue) on the *C*_3_ axis.

Following our investigation on the related dithiolates **1dtp** and **2dtp**,^19^ we also computed various possible isomers of **1** and **2**, all differing from the topology of their protecting outer shell made of 12 dsep ligands and 7 and 8 capping Ag(I) atoms, respectively. As for their dtp analogues, the free energy differences between the computed isomers are small, lying in a range of 4.2 and 8.3 kcal/mol in the cases of **1** and **2**, respectively (Figure 7). The most stable isomer of **1** (labeled *C*_3b_) is of *C*_3_ ideal symmetry (ligand configuration considered). Its Ag_12_@Ag_7_ silver framework can be described as being made of an icosahedron inscribed in a cube, of which one vertex is vacant. The encapsulated Pd–H unit is aligned along the *C*_3_ axis, which contains the missing vertex. This structure differs from the SCXRD one, which is also of *C*_3_ symmetry (labeled *C*_3a_ in Figure 7) but has a different arrangement of the seven outer metals (Figures 2 and 7) and is computed to be only 3.2 kcal/mol higher in free energy. Adding one supplementary Ag(I) capping atom on the *C*_3_ axis of these two structures generates two isomers of **2**, still of *C*_3_ symmetry, which differ in energy by only 1.3 kcal/mol. Alternative *C*_1_ structures generated from related species^19^ lie at somewhat higher energy (see Figure 7). The most stable isomer of **2** is of *C*_3_ symmetry (labeled *C*_3b_) and depicts a Ag_12_ icosahedron inscribed in a Ag_8_ cube, with the Pt–H unit along one cubic diagonal, which corresponds to the SCXRD structure of **2**. Hence, the encapsulated hydride lies within a PdAg_3_ tetrahedron, of which the three Ag atoms depict one of the eight icosahedron faces, which are capped by an outer Ag(I) atom. Moving this hydride inside one of the 20 PdAg_3_ tetrahedra, which are not capped by a Ag(I) atom, leads to an additional isomeric structure of *C*_1_ symmetry (labeled *C*_1d_), which is computed to be almost isoenergetic with the global C_3_ minimum (Figure 7). This result suggests that all the PdAg_3_ tetrahedral sites in **2** are statistically nearly equiprobable for H-occupation and that the encapsulated hydride is likely moving nearly freely from one to another.

**Figure 8:**
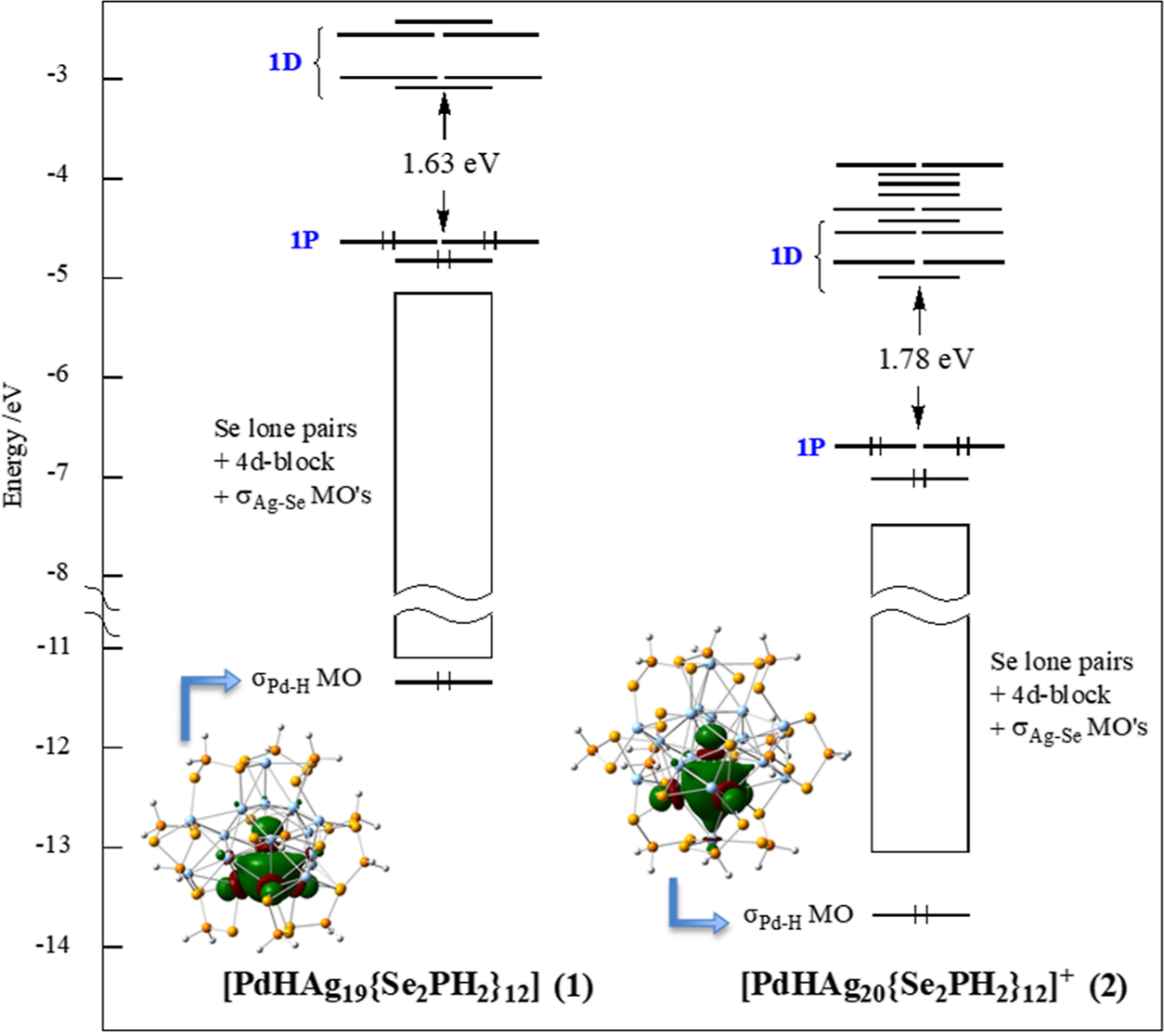
Kohn–Sham frontier orbital diagram of **1** and **2** [isomeric forms corresponding to their SCXRD structures (*C*_3a_ and *C*_3b_ for **1** and **2**, respectively)]. The 1P and 1D orbitals are plotted in Figure S10.

This is consistent with the VT ^1^H and ^31^P NMR data, which indicate a highly symmetrical cluster (on average) at the NMR time scale (see above). As also found for their dtp analogues,^19^ the various isomers computed for **1** and **2** exhibit similar metrics and electronic structure. The Kohn–Sham orbital diagrams of the *C*_3_ geometries that correspond to their SCXRD structures are provided in Figure 8, and their 1P and 1D orbitals are plotted in Figure S9. As their dtp analogues, they can be described as 8-electron *superatoms* containing an icosahedral [(PdH)@Ag_12_]^5+^ core with 1S^2^ 1P^6^ 1D^0^ configuration. In both cases, the 1P and 1D levels constitute the cluster frontier orbitals (Figures 8 and S9). Whereas the electron of the encapsulated hydrogen is part of the 8-electron count, its 1s orbital is barely involved in the construction of the *superatomic* orbitals. Rather, it interacts preferentially with the 4d_*z*^2^_ orbital of Pd (assuming *z*//Pd–H), making a fairly localized Pd–H bond. The same situation was found for the related dtp species.^19^

The time-dependent DFT (TDDFT)-simulated UV–vis spectra of **1** and **2**, considered in their *C*_3_ SCXRD configurations, were simulated at the CAM-B3LYP/Def2-TZVP level (see Computational Details) and are shown in Figure S10. They exhibit the same double band shape as their experimental counterparts (Figure 6), although the respective intensities of the two bands are switched in the case of **1**. In the simulated spectra, the highest energy band is centered at 364 and 373 nm for **1** and **2**, respectively, and the low energy band is at 487 and 453 nm. These values are in satisfying agreement with the experiment. As in the case of the dtp analogues,^19^ the two peaks are of 1P → 1D nature, being the result of a single-band splitting caused by the lowering of spheroidal symmetry induced by the presence of an encapsulated hydrogen atom. The DFT-simulated phosphorescence emission spectrum of **2**, which accounts for vibronic transitions (see Computational Details), is shown in Figure 6. Its peak at 764 nm is in good agreement with the experimental value of 739 nm. The emissive triplet state can be described as resulting from an electron transfer from the doubly degenerate 1P_*x*,*y*_ HOMO to the 1D_*z*^2^_ LUMO (Figure 8).

### Antibacterial Activity

The combined series of dsep and dtp analogues for 8-electron superatomically doped clusters provides an opportunity to evaluate their antibacterial activity. The utilization of silver for antimicrobial purposes predates the advent of pharmaceutical antibiotics. Furthermore, commercializing silver nanoparticles for antimicrobial activity has become a common practice. Several publications have reported the viability of silver and a combination of stabilizing agents as viable candidates for antibacterial activity.^29−31^

**Figure 9:**
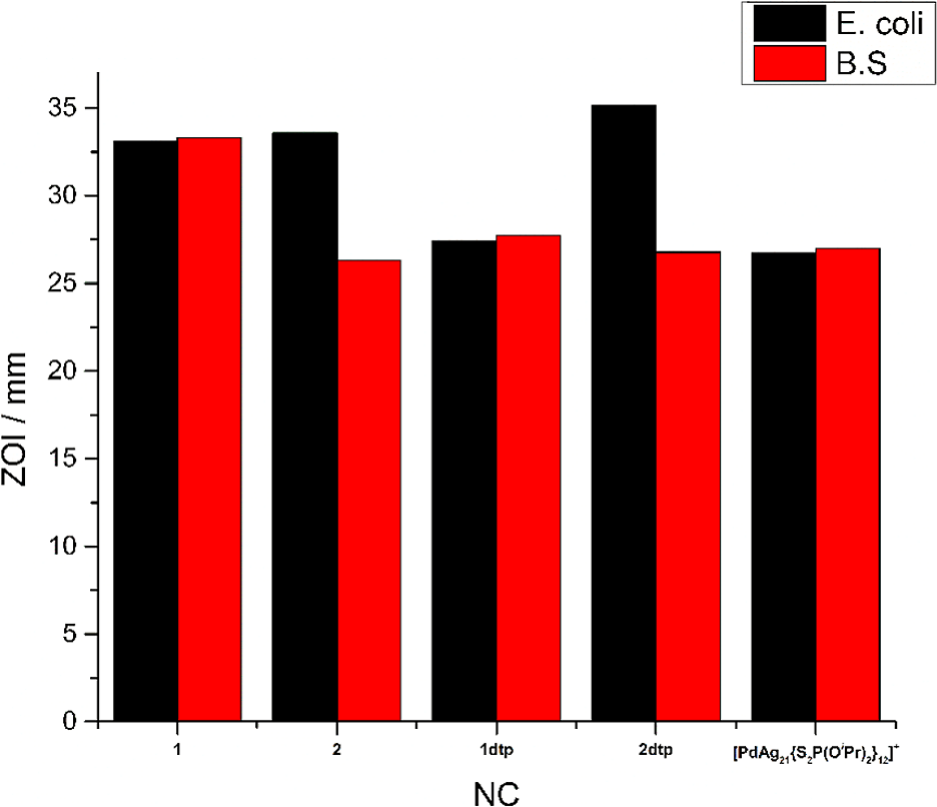
Assessment of the antibacterial properties of a series of Pd/Ag superatomic NC.

The bimetallic system Pd/Ag has also recently been the subject of antibacterial activity in nanoparticle form.^32,33^ However, it should be noted that studies involving atomically precise NC, which have fully identified structural features, are exceedingly rare in the literature. The critical factor to the antibacterial activity has been the ability to release Ag^+^ at a specific rate and location.^34^ Intuitively, using atomically precise constructs for such an application should be ideal since these assemblies lie at the junction of bulk NCs and precise molecular constructs. The ability to precisely modify the surface and size of the NCs allows engineering of NCs for particular cell membranes or environments. In this preliminary investigation, we chose two model bacteria, *Escherichia coli* (*E. coli*) and *Bacillus subtilis* (*B. subtilis*), and performed a zone of inhibition (ZOI) assay to determine the comparative antibacterial activity of each NC and their dtp analogues.^35^ The assay results are summarized in Figure 9 and Table S2. To provide a basis for comparison, several related NCs and ligands are tested under the same conditions, Table S2. The ligands dtp and dsep also have antibacterial properties, contributing to the NCs’ effectiveness. Notably, the monometallic {Ag_20_[Se_2_P(O^*i*^Pr)_2_]_12_} has superior performance than bulk nanoparticles prepared from traditional wet methods.^36^ The performance is comparable to Ag nanoparticles, usually derived from a combination of silver precursors with natural extracts^37−39^ and Ag composites.^40,41^ The inclusion of Pd/PdH into the kernel results in a marked increase in the antibacterial efficacy, ca. 2.5-fold increase in the ZOI over {Ag_20_[Se_2_P(O^*i*^Pr)_2_]_12_}. The NC does not outperform the commercial antibiotic ciprofloxacin. However, knowledge of the atomically precise structure provides a robust platform for future structural investigation into the complex interactions in these systems.

## Conclusions

An expansion of a series of superatomically Pd-doped silver NCs has been achieved by controlled transformation of the passivating shell. The dsep ligands profoundly affect both of the NC structures, which in turn greatly influences their properties. VT-NMR demonstrates the rigidity of the dsep ligand, with only one configurational isomer existing at low temperatures. Importantly, these isomers directly correspond to the metallic configurations observed in the solid state. The stability afforded by the dsep ligands contrasts with the complex isomeric distribution observed for the dtp relatives in solution and the dtp’s inability to stabilize the most symmetrical arrangements in the solid state. The ligand nature heavily influences the optical properties, and the 12 dsep ligands surrounding the metallic framework induce relatively similar absorption profiles for **1**–**2**. The shorter wavelength emissions come at the cost of reduced lifetimes. However, the ability to move an emission wavelength a few nanometers with a precise modification will be crucial for future applications. The complete series showed antibacterial activity against *E. coli* and *B. subtilis*. Although this is not a commercially viable system for antibacterial activity, we hope such information provides a basis for future investigations into this avenue of research.

## Experimental Section

### Materials and Characterization

Commercially sourced sodium borohydride (NaBH_4_, 98%), HTFA (99.5%), and sodium borodeuteride (NaBD_4_, 98%) were applied without further purification. {PdHAg_19_[S_2_P(O^*i*^Pr)_2_]_12_}^19^ and NH_4_[Se_2_P(O^*i*^Pr)_2_]^42^ were prepared following the method outlined in the literature. These reagents are considered corrosive and should be used with caution. All reactions were carried out under an inert atmosphere using standard Schlenk line techniques. ^1^H (400 MHz), ^31^P{^1^H} (161.97 MHz), and ^2^H (61.42 MHz) NMR spectra were recorded on a Bruker Advance II 400 MHz spectrometer. VT-^31^P{^1^H} (242.94 MHz) and VT-^1^H (600 MHz) NMR spectra were recorded on a Bruker AV-600 BBO probe spectrometer. ^19^F NMR (470.59 MHz) spectrum was recorded on the AVIIIHD-500 BBFO probe. The chemical shift (δ) and coupling constants (*J*) are reported in parts per million and Hz, respectively. ESI-MS spectra were captured on the AB SCIE X QSTAR XL high-resolution electrospray mass spectrometer. At 298 K, the Agilent Cary-60 and PerkinElmer Lambda 750 UV–visible spectrometers were used to detect the optical absorption spectra. On an Edinburgh FLS920 fluorescence spectrometer, the lifetime decays and PL spectra were monitored in an EPR tube with a liquid cryogenic system at 77 K. A PHI 5000 VersaProbe scanning ESCA microprobe on an X-ray photoelectron spectrometer was used to record the XPS spectra.

### Synthesis of {PdHAg_19_[Se_2_P(O^*i*^Pr)_2_]_12_}, **1**

{PdHAg_19_[S_2_P(O^*i*^Pr)_2_]_12_} (0.01 g, 0.002 mmol) and NH_4_[Se_2_P(O^*i*^Pr)_2_] (0.0078 g, 0.07 mmol) were dissolved in THF (15 mL). The brown solution was stirred at room temperature for 10 min, resulting in a mulberry-colored solution. The solvent is removed in vacuo to obtain a residue of NH_4_[S_2_P(O^*i*^Pr)_2_] and **1**. The crude product was purified by column chromatography (alumina stationary phase), and **1** was eluted with diethyl ether in 54% yield. ^1^H NMR (600 MHz, CD_2_Cl_2_, δ, ppm, 293 K): 4.99 (septet, CH, 24H), 1.43 (d, CH_2_, 144H); −7.18 (^1^*J*_HAg_ = 10.35 Hz). ^31^P{^1^H} NMR (242.94 MHz, CD_2_Cl_2_, δ, ppm, 293 K): 63.63 (^1^*J*_PSe_ = 633.36 Hz). ESI-MS (*m*/*z*): exp. 5949.8566 Da (calc. 5949.9058 Da for [**1** + Ag]^+^). UV–vis [λ_max_ in nm, (ε in M^–1^ cm^–1^)]: 414 (41,134), 500 (26,042). Elemental Anal. C_72_H_169_Ag_19_O_24_P_12_PdSe_24_ [calc.: C %, 14.8 and H %, 2.92]: C %, 14.39, and H %, 2.77.

(**1**_**D**_): The deuteride analogue was prepared under the same conditions with the parent cluster substituted for the deuterated analogue {PdDAg_19_[S_2_P(O^*i*^Pr)_2_]_12_}, yield 71%. ^2^H NMR (61.42 MHz, CHCl_3_, δ, ppm): −7.11. ESI-MS (*m*/*z*): exp. 5950.0186 Da (calc. 5950.8991 Da for [**1**_**D**_ + Ag]^+^).

### Synthesis of {PdHAg_20_[Se_2_P(O^*i*^Pr)_2_]_12_}CF_3_COO, **2**

A solution of {PdHAg_19_[Se_2_P(O^*i*^Pr)_2_]_12_} (0.03 g, 0.005 mmol) in hexane (50 mL) is treated with 0.4 μL of HTFA at ambient temperature. After stirring for 15 min, the reaction mixture was allowed to stand for 20 min, resulting in the separation of an orange precipitate from a colorless solvent. The precipitate is consolidated and washed with hexane, yield: 62%. ^1^H NMR (600 MHz, CD_2_Cl_2_, δ, ppm, 293 K): 4.97 (septet, CH, 24H), 1.44 (d, CH_2_, 144H); −10.70 (^1^*J*_HAg_ = 9.70 Hz). ^31^P{^1^H} NMR (242.94 MHz, CD_2_Cl_2_, δ, ppm, 293 K): 63.26 (^1^*J*_PSe_ = 635.44 Hz). VT-^31^P{^1^H} NMR (242.94 MHz, CD_2_Cl_2_, δ, ppm, 213 K): 64.88 (^1^*J*_PSe_ = 579.47 and 684.06 Hz). ^19^F NMR (470.59 MHz, CD_2_Cl_2_, δ, ppm, 298 K): −76.50. ESI-MS (*m*/*z*): exp. 5949.8784 Da (calc. 5949.9058 Da for [**2**]^+^). UV–vis [λ_max_ in nm, (ε in M^–1^ cm^–1^)]: 408 (401,294), 486 (225,676). Elemental Anal. C_74_H_169_Ag_20_F_3_O_26_P_12_PdSe_24_ [calc.: C %, 14.66; H %, 2.81]: C %, 14.65; H %, 2.73.

(**2**_**D**_): The deuterated analogue was prepared under the same conditions with the precursor cluster substituted for the deuterated analogue {PdDAg_19_[S_2_P(O^*i*^Pr)_2_]_12_}, yield: 73%. ^2^H NMR (61.42 MHz, CHCl_3_, δ, ppm): −10.637. ESI-MS (*m*/*z*): exp. 5950.8874 Da (calc. 5950.8991 Da for [**2**_**D**_ + Ag]^+^).

### Computational Details

Geometry optimizations were carried out within the DFT formalism with the Gaussian 16 package,^43^ using the BP86 functional^44,45^ and the Def2-TZVP basis set.^46,47^ All of the optimized geometries were computed without any symmetry constraint and characterized as true minima by vibrational analysis. The natural atomic orbital (NAO) charges and Wiberg bond indices were computed with the NBO 6.0 program.^48^ The UV–visible transitions were calculated on the above-mentioned optimized geometries by means of TDDFT calculations, with the CAM-B3LYP functional^49^ and the Def2-TZVP basis set. The UV–visible spectra were simulated from the computed TDDFT transitions and their oscillator strengths by using the SWizard program,^50^ each transition being associated with a Gaussian function of half-height width equal to 2000 cm^–1^.

The phosphorescence emission spectra were simulated from DFT calculations on the optimized singlet ground and first excited triplet states using the Adiabatic Hessian method within the Franck–Condon principle, which accounts for vibrational mode mixing and a proper description of both optimized ground and excited state potential energy surfaces. The triplet state was optimized at the unrestricted BP86/Def2-TZVP level (assuming the corresponding singlet structure was the starting geometry) and characterized as a true minimum. The lowest normal modes in the vibronic treatment were neglected in order to obtain sufficient spectrum progression. We employed class-based prescreening to limit the number of terms involved in the vibronic calculation with the following settings: C_1_^max^ = 70, C_2_^max^ = 70, and N_1_^max^ = 100 × 10^8^.^51,52^ The vibronic plot was realized using the VMS software.^53^

### X-ray Crystallography

Single crystals were covered in paratone, mounted on a glass fiber, and cooled in a nitrogen cold stream to 100 K. Data were collected on a Bruker APEX II CCD diffractometer using graphite monochromated Mo Kα radiation (λ = 0.71073 Å). Absorption corrections were performed with SADABS,^54^ and the integration of raw data frames was done with SAINT.^55^ The structures were solved by direct methods and refined by least squares against *F*^2^ using the SHELXL-2018/3 package,^56^ incorporated in SHELXTL/PC V6.14.^57^ All non-hydrogen atoms were refined anisotropically.

## Supporting Information Available

NMR spectra, ESI-MS spectra, X-ray crystallography refinement parameters, and *xyz* coordinate file (PDF)

## Abbreviations

NC: atomically precise nanocluster
dsep: diselenophosphate
dtp: dithiophosphate
LEIST: ligand exchange-induced structure transformation
