# Supplementary material for: Diselenophosphate Ligands as a Surface Engineering Tool in PdH-Doped Silver Superatomic Nanoclusters
Source: Inorg Chem. 2024 Jan 22;63(5):2766–75. doi: 10.1021/acs.inorgchem.3c04253 (PMC10848256; doi:10.1021/acs.inorgchem.3c04253)
Supplement: Supplementary file 1 — ic3c04253_si_001.pdf [file ic3c04253_si_001.pdf]

Supporting Information

Diselenophosphate ligands as a surface engineering tool in PdH doped silver superatomic nanoclusters

Yu-Rong Ni,[a] Michael N. Pillay,[a] Tzu-Hao Chiu,[a] Jagadeesh Rajaram,[a]

Ying-Yann Wu,[a] Samia Kahlal,[b] and Jean-Yves Saillard,[b] and C. W. Liu*[a]*

a. Department of Chemistry, National Dong Hwa University, Hualien, 97401, Taiwan, Republic of China. E-mail: chenwei@gms.ndhu.edu.tw
b. Univ Rennes, CNRS, ISCR-UMR 6226, F-35000, France.
Email: jean-yves.saillard@univ-rennes.fr

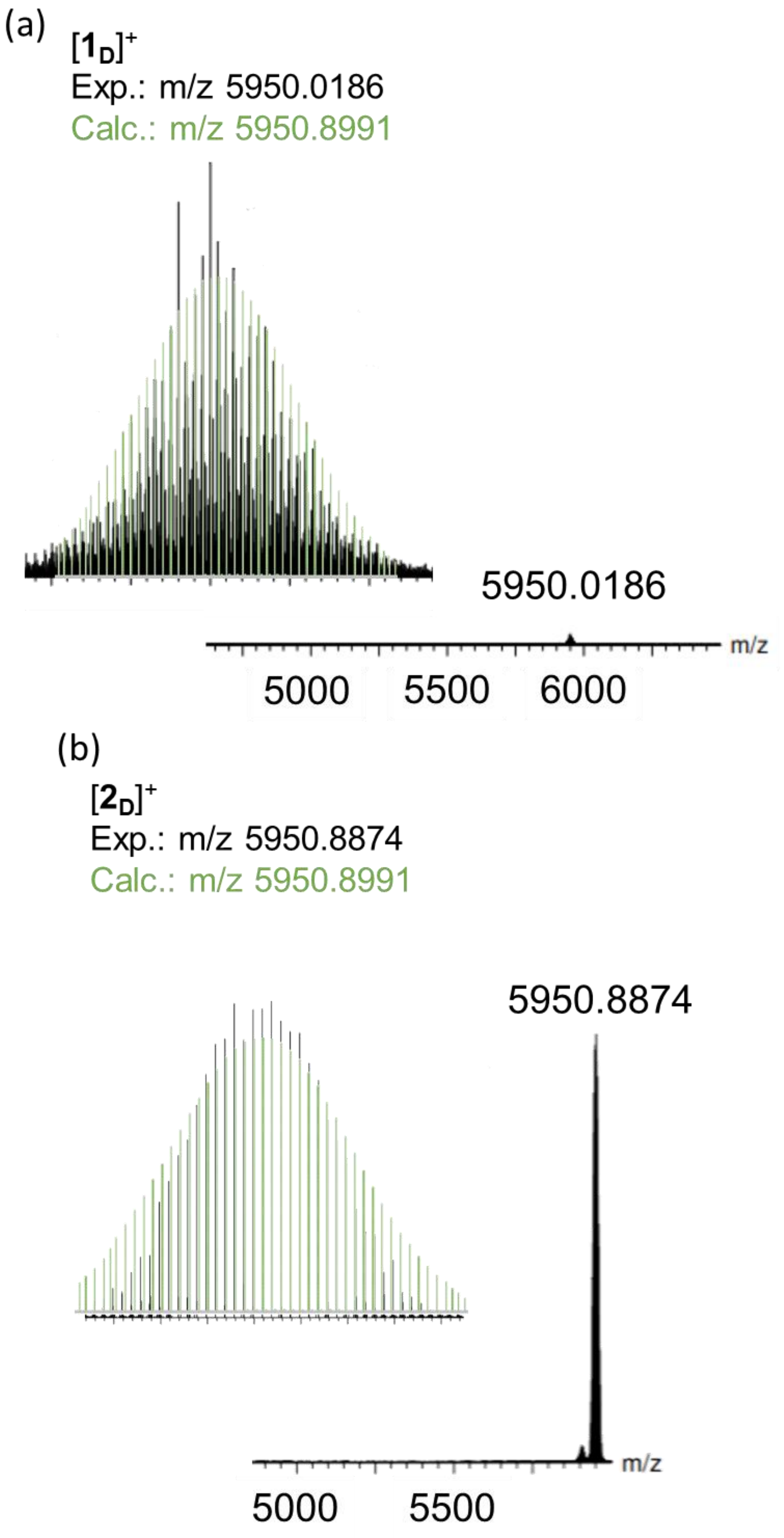

Figure S1. ESI-TOF-MS spectrums of deuteride analogs for (a) $\mathbf{1_D}$, (b) $\mathbf{2_D}$.

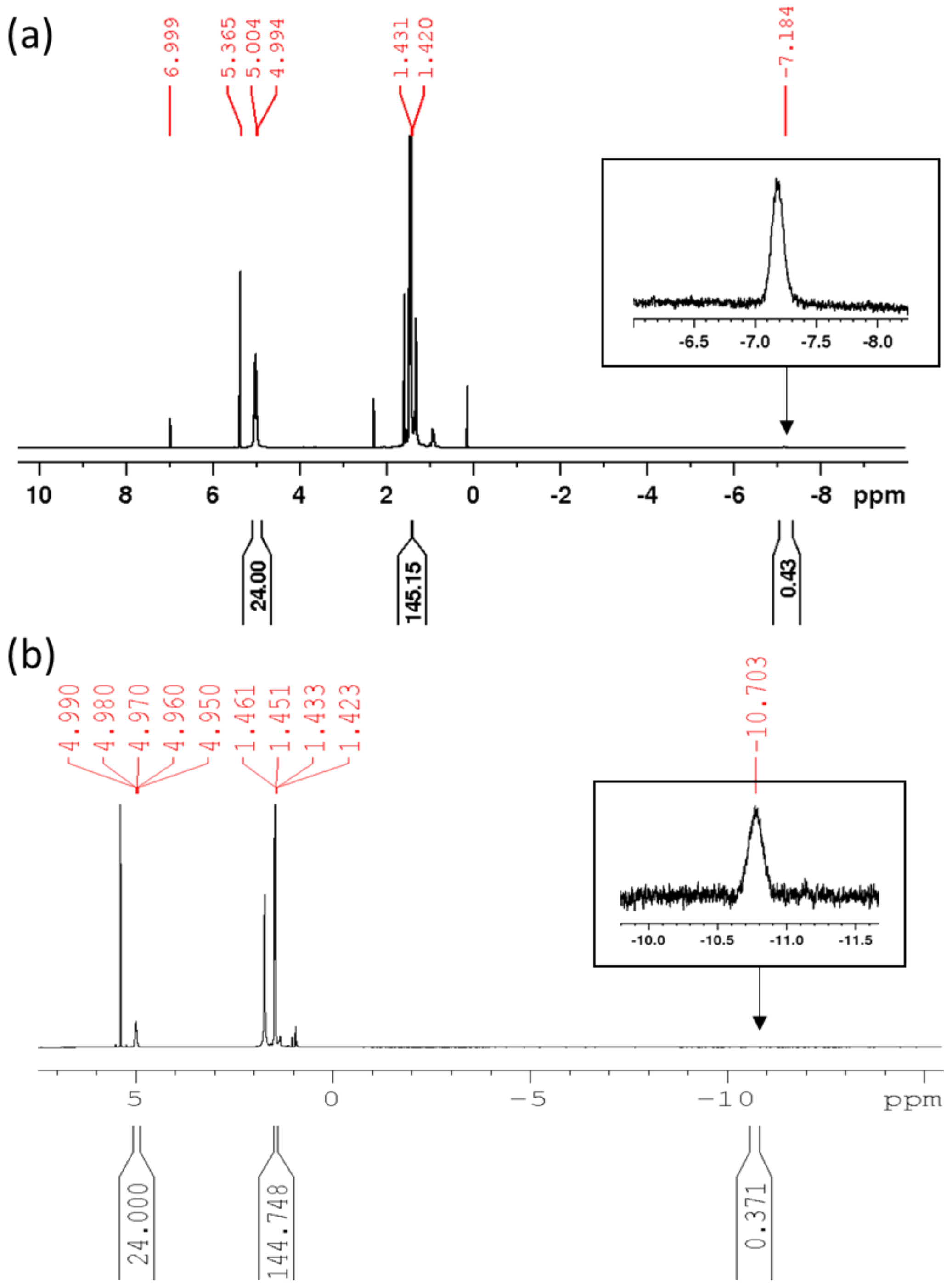

Figure S2. ^{1}H NMR spectrum (600 MHz) of (a) **1** and (b) **2** in CD_2Cl_2 at 293 K.

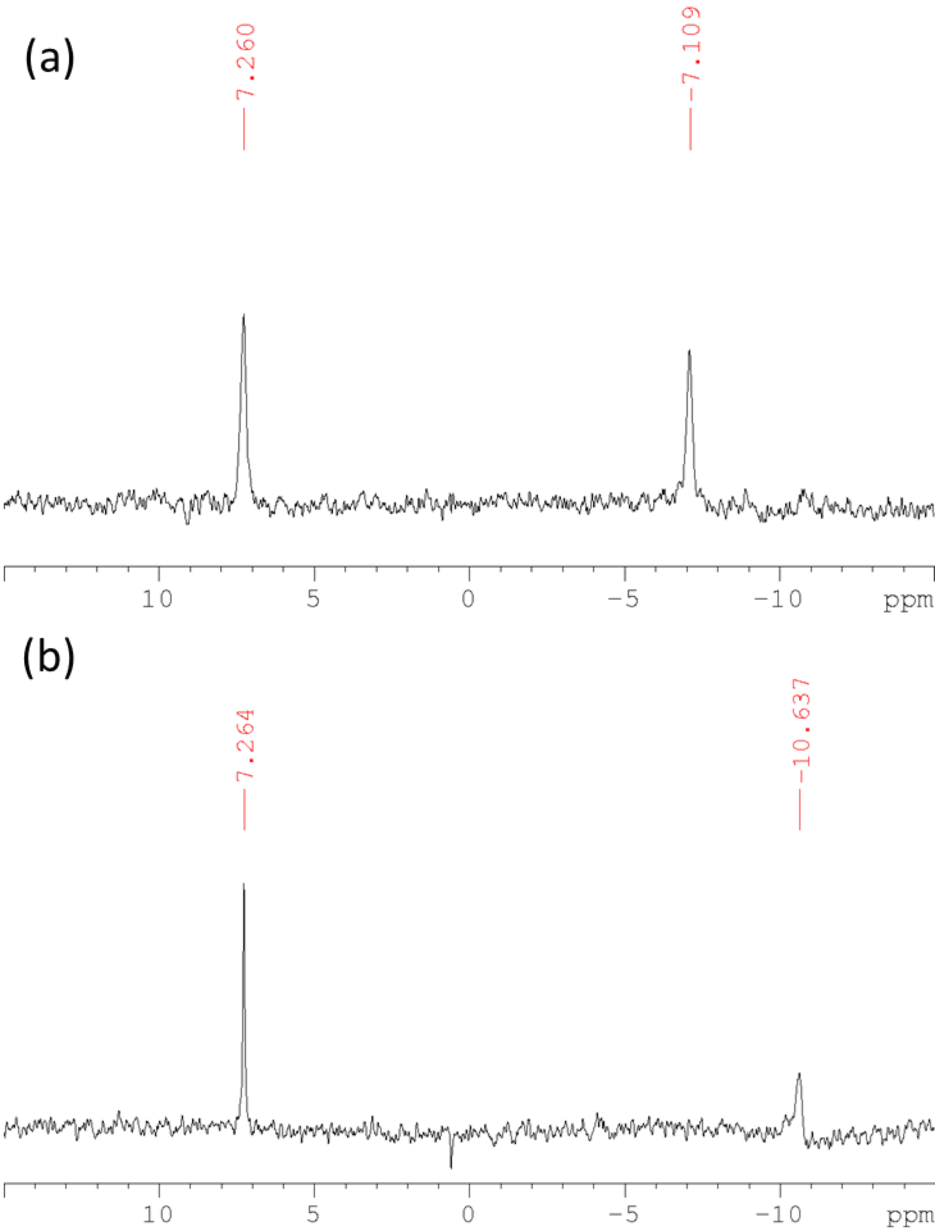

Figure S3. ^{2}H NMR spectrum (61.42 MHz) of (a) **1** and (b) **2** in $CDCl_3$.

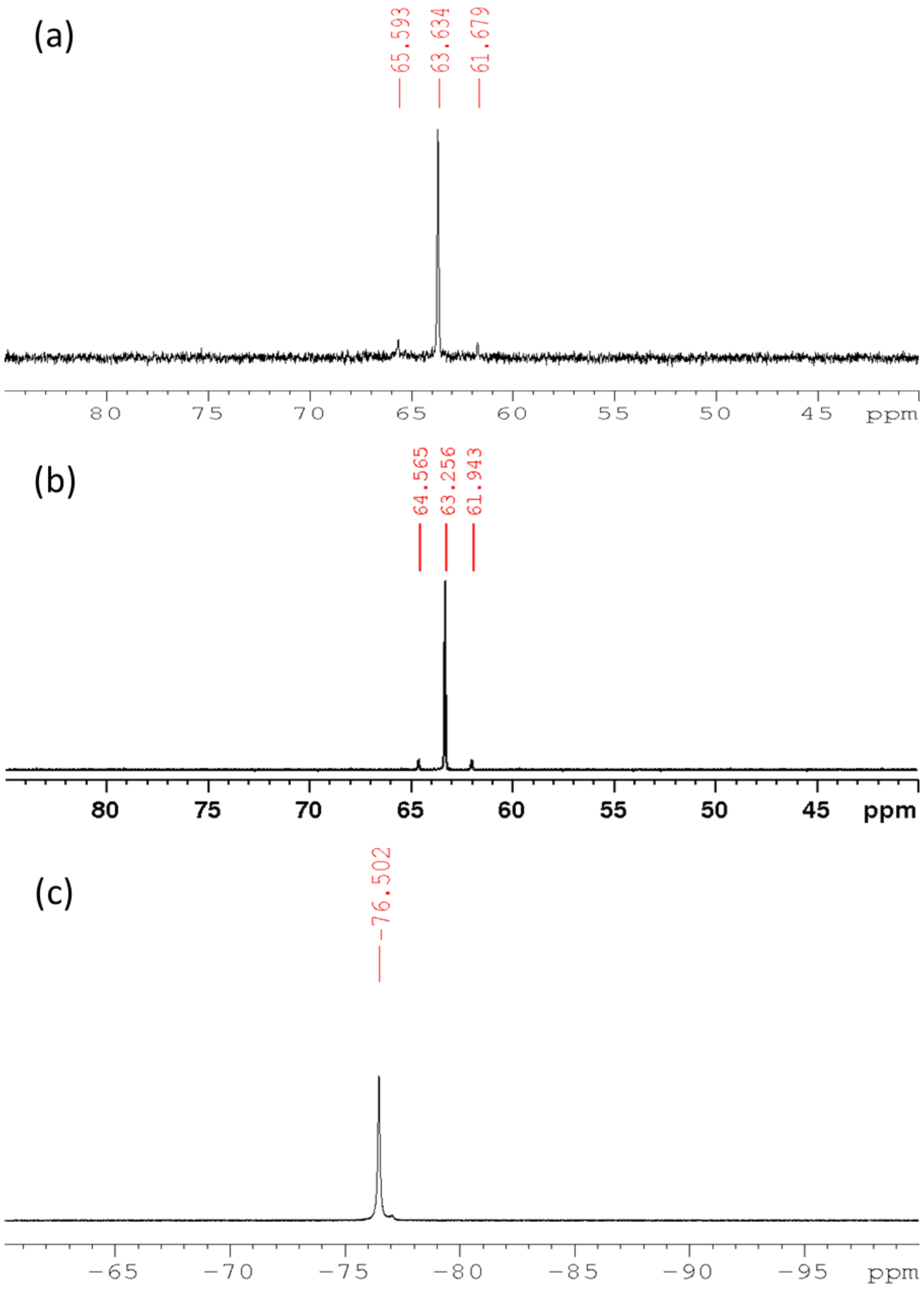

Figure S4. $^{31}P\ \{^{1}H\}$ NMR spectrum (242.94 MHz) of (a) **1** ($^{1}J_{PSe}$ = 633.36 Hz) and (b) **2** ($^{1}J_{PSe}$ = 635.44 Hz) in $CDCl_3$ at 293 K. (c) ^{19}F NMR spectrum (470.59 MHz) of **2** in CD_2Cl_2.

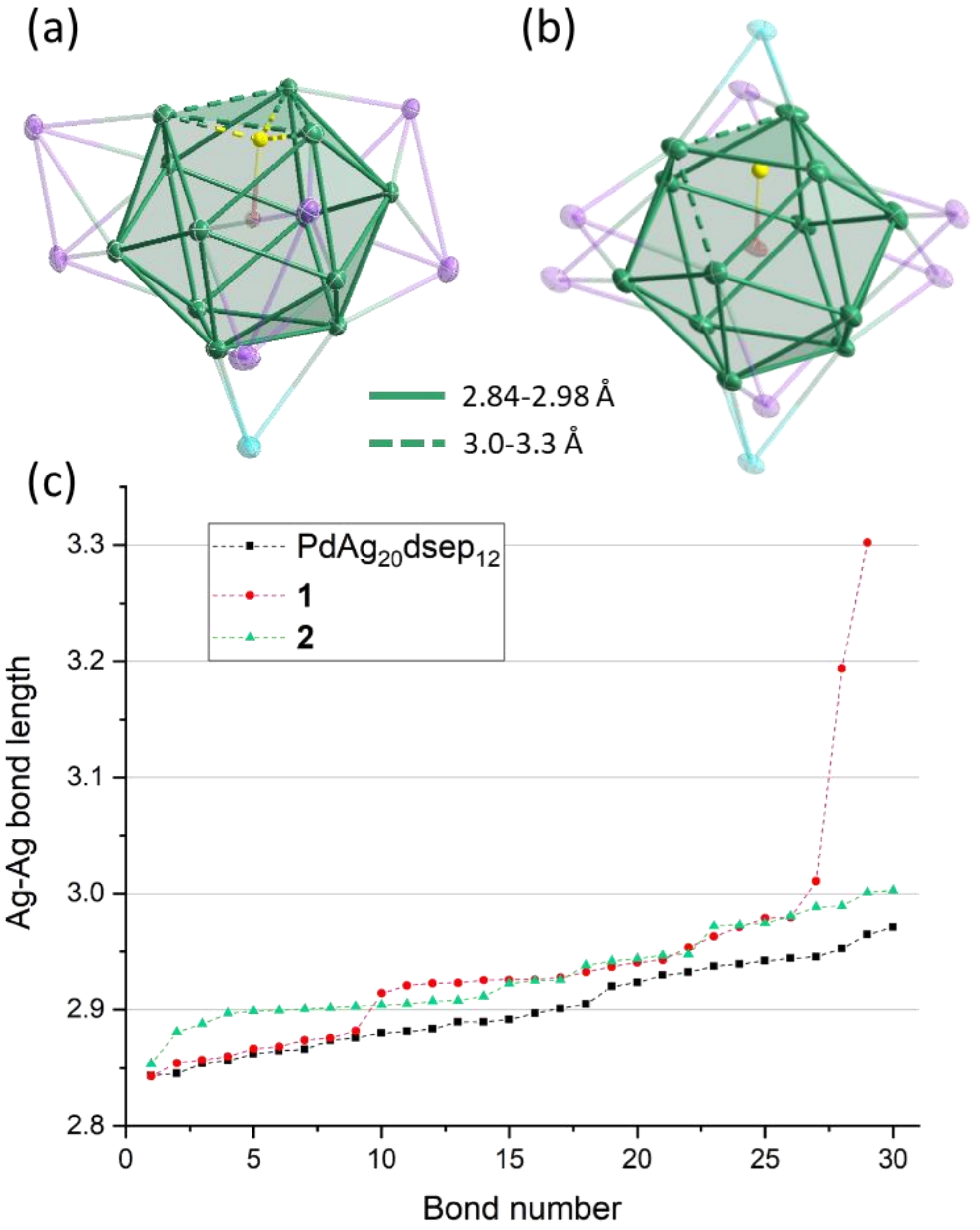

Figure S5. Metallic framework of (a) **1**, and (b) **2**. (c) The Ag-Ag bond lengths of Ag_{12} icosahedron comparison chart.

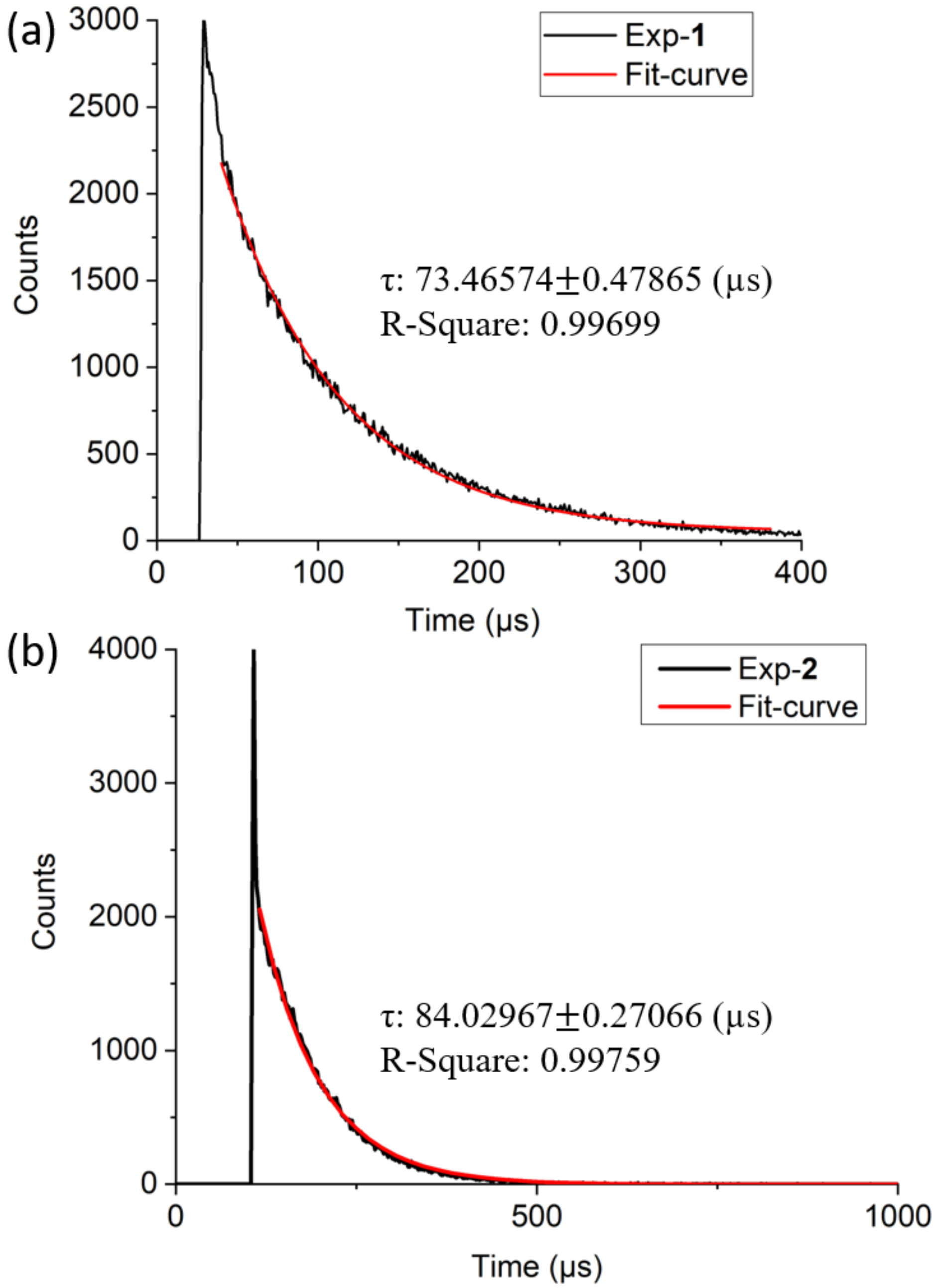

Figure S6. Photoluminescence lifetime decay spectrum of (a) **1**, and (b) **2**.

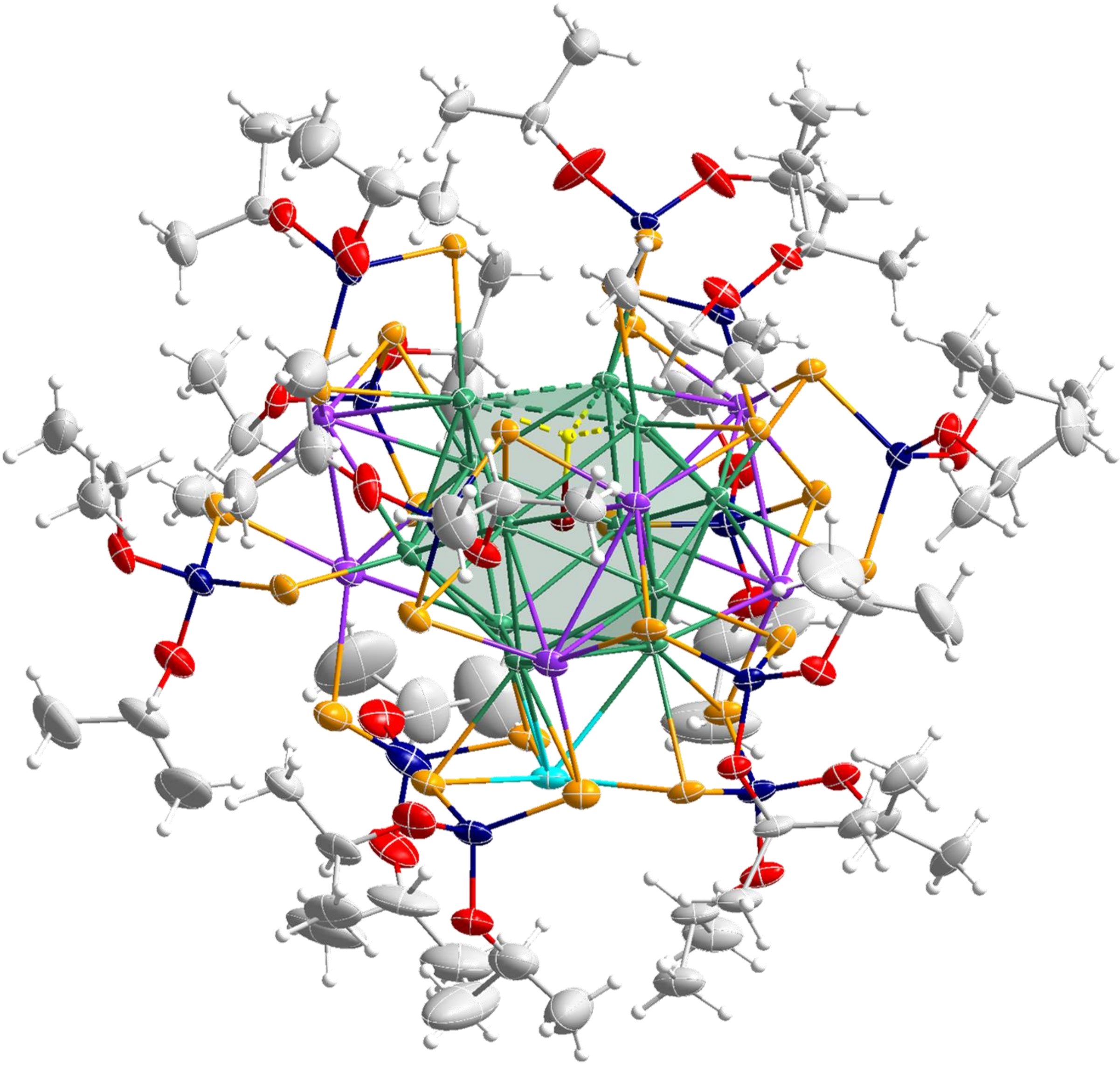

Figure S7. The X-ray structure of **1.**

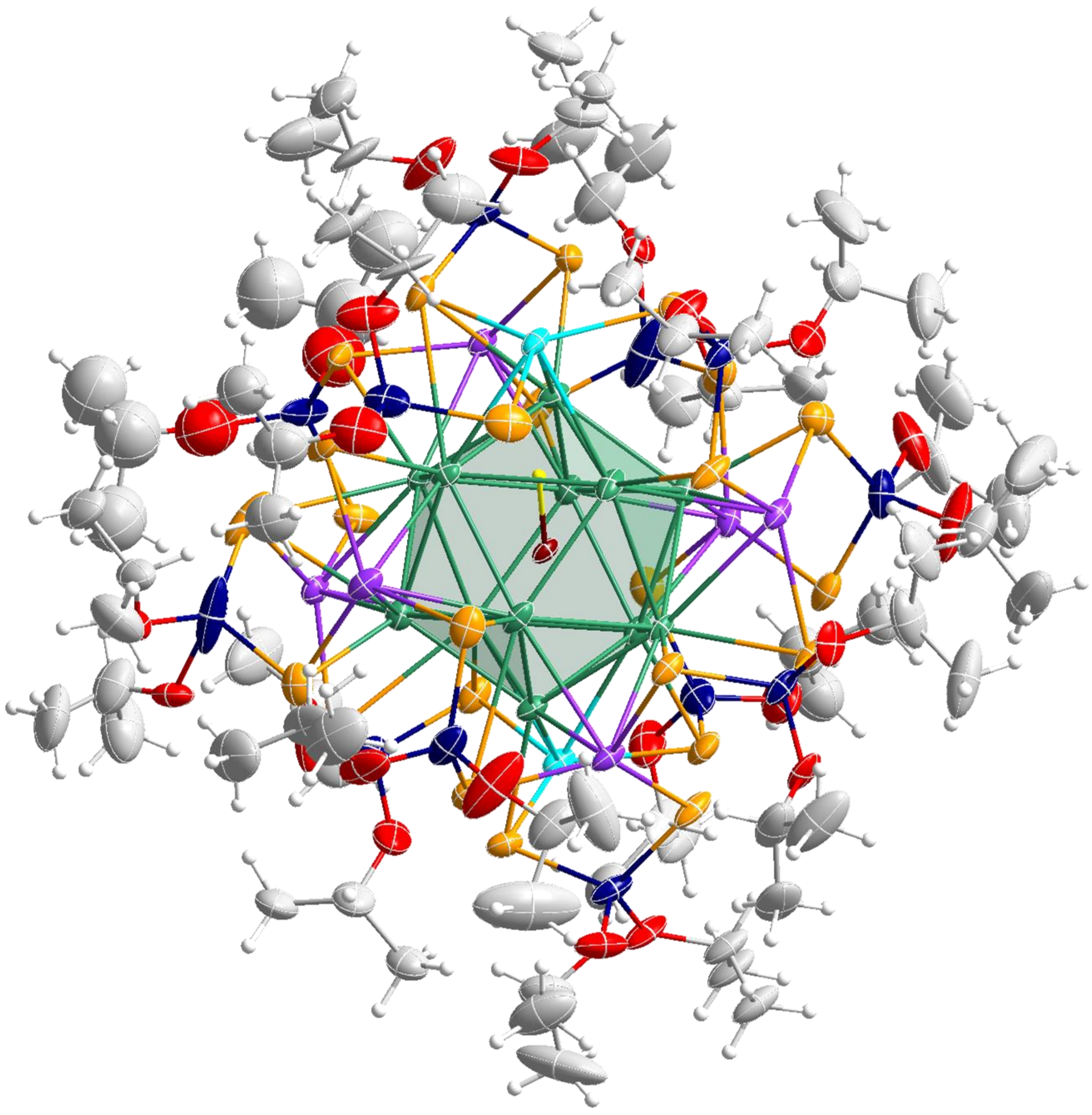

Figure S8. The X-ray structure of **2.** Thermal ellipsoid drawn at 35% except H.

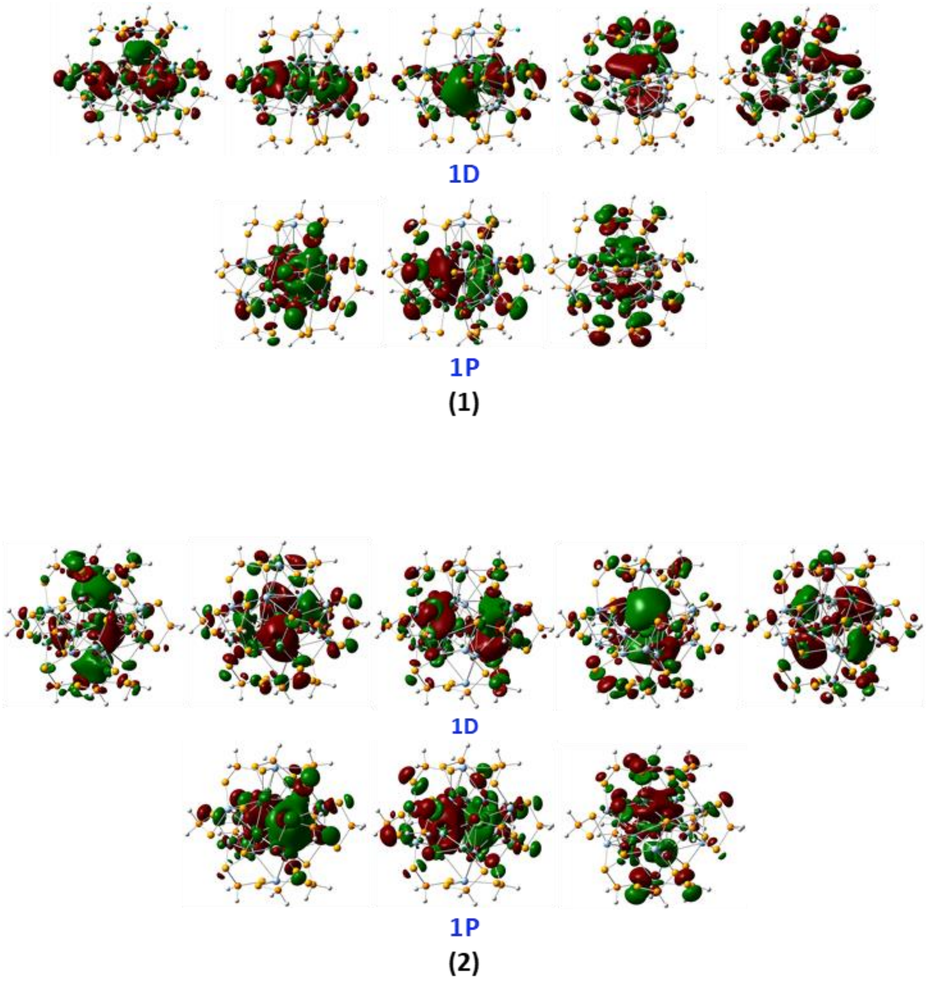

Figure S9. Kohn-Sham frontier orbitals of **1** and **2** (isomeric forms corresponding to their SCXRD structures, see Figure 7). The corresponding orbital energy diagram is shown in Figure 8.

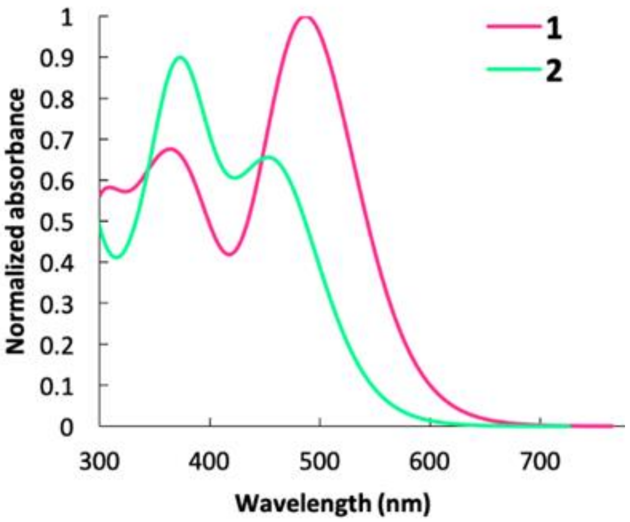

Figure S10. The TD-DFT simulated UV-vis spectra of **1** and **2** (isomeric forms corresponding to their SCXRD structures (C_{3a} and C_{3b} for **1** and **2**, respectively)).

Table S1. Crystal data and refinement of **1** and **2**.

Compound	[Pd(H)Ag_{19}{$Se_2P(O^iPr)_2$}$_{12}$], 1	[Pd(H)Ag_{20}{$Se_2P(O^iPr)_2$}$_{12}$], 2
Chemical formula	$C_{72}H_{169}Ag_{19}O_{24}P_{12}PdSe_{24}$	$C_{72}H_{168}Ag_{20}O_{24}P_{12}PdSe_{24}$
CCDC	2310294	2310295
Formula weight	5841.67	5948.53
Crystal system, space group	Monoclinic, *P2(1)/n*	Monoclinic, *P2/c*
a, Å	29.814(2)	16.8687(14)
b, Å	17.4351(2)	21.9917(16)
c, Å	33.995(2)	23.9103(19)
α, deg.	90	90
β, deg	113.269(2)	108.872(2)
γ, deg	90	90
Volume, $Å^3$	16233(2)	8393.2(11)
Z	4	2
ρcalcd, g·cm^{-3}	2.390	2.354
μ, mm^{-1}	7.887	7.740
Temperature, K	100(2) K	100(2) K
θmax, deg. / Completeness, %	26.437 / 99.9	24.998 / 99.9
Reflections collected / unique	152239 / 33289 [R(int) = 0.0531]	49137 / 14765 [R(int) = 0.0647]
Restraints/parameters	617 / 1537	388 / 824
R1a, wR2b [I > 2σ(I)]	R1 = 0.0455, wR2 = 0.1010	R1 = 0.0877, wR2 = 0.2149
R1a, wR2b (all data)	R1 = 0.0630, wR2 = 0.1094	R1 = 0.1197, wR2 = 0.2366
Goodness of fit	1.106	1.088
Largest diff. peak and hole, e/$Å^3$	2.162 and -1.431	3.242 and -1.798

$^{a}R1 = \Sigma \| |F_o| - |F_c| \| / \Sigma |F_o|$. $^{b}wR2 = \{ \Sigma[w(F_o^2 - F_c^2)^2] / \Sigma[w(F_o^2)_2] \}^{1/2}$

Table S2. Zone of inhibition data for **1**, **2** and related compounds.

Bacteria	Compound	Replicate			Average	SD	RSD %
		1	2	3			
E Coli.	$[PdHAg_{19}\{S_2P(OPr)_2\}_{12}]$	27,42	27,40	27,47	**27,43**	0,03	0,10
	$[PdHAg_{20}\{S_2P(OPr)_2\}_{12}](CF_3COO)$	35,04	35,25	35,10	**35,13**	0,09	0,25
	$[PdAg_{21}\{S_2P(OPr)_2\}_{12}](BF_4)$	26,89	26,29	26,93	**26,71**	0,29	1,10
	$[PdHAg_{19}\{Se_2P(OPr)_2\}_{12}]$ (**1**)	33,01	33,23	33,04	**33,09**	0,10	0,29
	$[PdHAg_{20}\{Se_2P(OPr)_2\}_{12}](CF_3COO)$ (**2**)	33,62	33,41	33,66	**33,56**	0,11	0,33
	$[Ag_{20}\{Se_2P(OPr)_2\}_{12}]$	13,77	12,52	13,53	**13,27**	0,54	4,08
	$NH_4[Se_2P(OPr)_2]$	13,44	13,51	12,53	**13,16**	0,45	3,38
	$NH_4[S_2P(OPr)_2]$	14,76	14,59	13,55	**14,30**	0,53	3,73
	Ciprofloracin	45,90	44,79	44,59	**45,09**	0,58	1,28
Bacillus subtilis	$[PdHAg_{19}\{S_2P(OPr)_2\}_{12}]$	27,72	27,77	27,68	**27,72**	0,04	0,13
	$[PdHAg_{20}\{S_2P(OPr)_2\}_{12}](CF_3COO)$	26,75	26,73	26,81	**26,77**	0,03	0,13
	$[PdAg_{21}\{S_2P(OPr)_2\}_{12}](BF_4)$	27,01	26,99	26,98	**26,99**	0,01	0,04
	$[PdHAg_{19}\{Se_2P(OPr)_2\}_{12}]$ (**1**)	33,30	33,27	33,33	**33,30**	0,03	0,08
	$[PdHAg_{20}\{Se_2P(OPr)_2\}_{12}](CF_3COO)$ (**2**)	26,05	26,74	26,08	**26,29**	0,32	1,20
	$[Ag_{20}\{Se_2P(OPr)_2\}_{12}]$	15,91	15,25	15,52	**15,56**	0,27	1,72
	$NH_4[Se_2P(OPr)_2]$	12,88	11,5	12,25	**12,21**	0,57	4,64
	$NH_4[S_2P(OPr)_2]$	11,09	11,24	10,5	**10,94**	0,32	2,92
	Ciprofloracin	50,54	49,09	51,1	**50,24**	0,49	0,96

Atomic coordinates (XYZ file below)

81

PdHAg19 C3a

Ag	0.607412	1.630218	2.065673
Ag	-1.716316	-0.286669	2.065159
Ag	1.105424	-1.340596	2.067048
Ag	2.776136	0.458330	0.383768
Ag	0.981431	2.620604	-0.694649
Ag	-1.785120	2.175713	0.380442
Ag	-2.759600	-0.461179	-0.695277
Ag	-0.991611	-2.633189	0.382724
Ag	1.779605	-2.159962	-0.692252
Ag	1.886818	0.329671	-2.478012
Ag	-1.227709	1.466903	-2.480550
Ag	-0.654952	-1.798876	-2.478883
Ag	-0.002859	0.002047	4.620142
Ag	-4.414974	1.018634	1.455806
Ag	-4.189845	2.079359	-1.721464
Ag	3.087678	3.314956	1.458956
Ag	3.899291	2.587547	-1.717237
Ag	1.325049	-4.331392	1.460706
Ag	0.294121	-4.670269	-1.716965
H	2.779167	-4.147150	5.020253
H	1.274997	-3.359539	6.389369
H	2.198352	4.486883	5.016641
H	2.267667	2.792005	6.387795
H	-3.555363	0.575145	6.385266
H	-4.987533	-0.333228	5.013827
H	-2.452464	-7.431733	0.351095
H	-1.692308	-6.782330	2.287267
H	-2.338829	-3.622863	-5.850873
H	-0.694905	-5.016122	-5.583581
H	5.347886	-4.667916	-1.520606
H	3.832034	-6.133447	-0.956029
H	4.697569	1.897048	-5.582403
H	4.311031	-0.223392	-5.846314
H	6.717957	1.928075	2.289917
H	7.662243	1.592490	0.355003

H	3.396982	6.383693	-0.961535
H	1.370483	6.963435	-1.528691
H	-5.210799	5.839542	0.344171
H	-5.030470	4.857227	2.280840
H	-3.992565	3.108967	-5.586711
H	-1.964534	3.837608	-5.853184
H	-6.715557	-2.298106	-1.525382
H	-7.226648	-0.252285	-0.961693
P	1.644233	-3.293508	5.019411
P	2.026723	3.077139	5.017341
P	-3.680818	0.222866	5.014992
P	-1.728055	-6.357537	0.932246
P	-1.211892	-3.821588	-5.007401
P	4.001990	-4.727482	-1.073497
P	3.920079	0.853810	-5.005058
P	6.369201	1.683371	0.934815
P	2.094383	5.828054	-1.079519
P	-4.643290	4.675303	0.926299
P	-2.699693	2.960358	-5.010032
P	-6.094191	-1.102824	-1.078066
Pd	0.000015	0.000116	-0.211284
Se	2.385734	-1.255618	4.565984
Se	0.027337	-4.138901	3.838123
Se	3.568332	2.098421	3.838352
Se	-0.108512	2.699343	4.562792
Se	-2.285985	-1.438112	4.563035
Se	-3.602885	2.046209	3.834371
Se	0.354749	-6.574455	0.207892
Se	-2.886166	-4.535878	0.727034
Se	-2.034172	-4.277177	-2.997760
Se	0.185756	-2.171928	-5.137278
Se	3.931191	-3.790735	0.901837
Se	2.724648	-3.945494	-2.690728
Se	4.723550	0.372347	-2.993827
Se	1.793116	1.241171	-5.137462
Se	5.370924	-0.230695	0.730811
Se	5.516292	3.594780	0.207607
Se	1.316723	5.300920	0.895813

Se	2.057066	4.328631	-2.694771
Se	-2.486385	4.767761	0.723456
Se	-5.871697	2.979646	0.201338
Se	-1.968120	0.925737	-5.139662
Se	-2.684657	3.900040	-3.000209
Se	-5.249020	-1.509317	0.897937
Se	-4.777083	-0.388319	-2.694651
H	0.000827	-0.000515	-1.920888

81

PdHAg19 C3b

Pd	-0.000516	-0.001358	-0.223094
Ag	1.504462	-0.868830	2.052397
Ag	-2.400705	1.442376	0.338639
Ag	-0.004354	1.734013	2.055314
Ag	-0.050714	-2.803345	0.338296
Ag	-1.503229	-0.873256	2.053120
Ag	2.450575	1.356383	0.340738
Ag	-1.701568	0.760345	-2.564243
Ag	2.414279	-1.461253	-0.680327
Ag	0.187043	-1.849544	-2.553115
Ag	0.061760	2.817828	-0.679455
Ag	1.509732	1.082726	-2.559934
Ag	-2.473367	-1.358449	-0.680418
Ag	-0.000675	-0.008928	4.615866
Ag	2.618075	-3.639955	1.461921
Ag	-4.457960	-0.450858	1.470258
Ag	1.840003	4.080121	1.476405
Ag	-2.641054	3.748836	-1.705147
Ag	-1.920977	-4.156096	-1.725927
Ag	4.564578	0.421108	-1.712396
H	-0.005661	0.005670	-1.913902
Se	2.593839	-0.728488	4.620083
Se	-0.676008	2.596958	4.626707
Se	-1.919374	-1.897843	4.618811
Se	1.061786	-4.082722	3.651179
Se	4.882861	-2.189579	1.817705
Se	2.279713	-5.249854	-0.691206

Se	-4.059157	1.109407	3.665359
Se	-0.547326	5.316556	1.836092
Se	-3.935211	3.765822	0.664986
Se	4.710482	-2.270206	-1.988805
Se	-1.292888	-5.298117	0.639490
Se	-4.330173	-3.138281	1.818396
Se	2.996469	2.946662	3.666005
Se	5.231276	1.522822	0.661622
Se	-0.381123	5.219212	-1.971000
Se	-4.327155	-2.937280	-1.987697
Se	-3.498746	2.373399	-3.833790
Se	0.219585	2.915164	-4.615755
Se	3.411237	4.596818	-0.669266
Se	-5.686474	0.656102	-0.675405
Se	-2.638661	-1.246920	-4.627513
Se	-0.297777	-4.191486	-3.855183
Se	2.407884	-1.623900	-4.629727
Se	3.793159	1.863070	-3.831288
P	2.290738	-2.895008	4.994172
P	5.778068	-2.732573	-0.093281
P	0.575015	-6.354005	0.083739
P	-5.253099	-3.638687	-0.091051
P	1.351025	3.417109	5.006065
P	5.216032	3.669863	0.111659
P	-0.519992	6.368710	-0.071676
P	-3.643634	-0.553944	5.001205
P	-5.786361	2.681467	0.108296
P	-1.830160	2.731173	-5.271751
P	-1.438352	-2.916719	-5.288591
P	3.275941	0.244620	-5.277909
H	6.089307	-4.117783	-0.140771
H	7.084629	-2.177624	-0.141392
H	3.591842	-3.448153	5.127624
H	1.800920	-3.059508	6.316768
H	0.928175	-7.119437	1.226698
H	0.261726	-7.390644	-0.834660
H	-0.451096	-2.510102	-6.224212
H	-2.220469	-3.795963	-6.086883

H	4.439437	0.001631	-6.058633
H	-6.609566	-3.218894	-0.133025
H	-5.422504	-5.047942	-0.142650
H	-6.623682	2.754476	1.253099
H	-6.528351	3.475715	-0.805412
H	-4.772868	-1.404920	5.132813
H	-3.539212	-0.053047	6.325763
H	-1.981353	1.684864	-6.219328
H	-2.197640	3.859387	-6.055486
H	-1.654135	7.222343	-0.120664
H	0.523665	7.331522	-0.113591
H	1.179545	4.820451	5.139832
H	1.735185	3.074298	6.329380
H	5.697353	4.355541	1.258456
H	6.274741	3.918721	-0.801275
H	2.448580	0.901595	-6.226235

81

PdHAg19 C1

Ag	0.160467	-2.554593	1.188521
Ag	2.503180	-0.949205	0.178272
Ag	1.254288	-0.138524	2.765807
Ag	-2.037047	-0.724241	2.320672
Ag	-2.244945	-1.816413	-0.460581
Ag	0.411071	-1.982694	-1.741338
Ag	1.506301	0.748774	-2.057468
Ag	1.963395	1.959745	0.676329
Ag	-0.742356	2.329732	1.958374
Ag	-2.827818	1.088498	0.038215
Ag	-0.433765	2.679565	-1.005015
Ag	-1.433984	0.267020	-2.419753
Ag	2.933070	-2.797332	2.582577
Ag	2.272470	-3.975738	-0.412680
Ag	2.407665	3.666354	-1.795031
Ag	0.786939	4.853375	0.849622
Ag	-4.884793	-1.064592	1.089010
Ag	-4.471071	-0.440794	-2.156584
Ag	3.480709	-1.801391	-2.519066

Se	1.037030	-0.555046	5.496277
Se	4.137592	-0.513478	3.409232
Se	0.744461	-3.819368	3.773337
Se	0.108896	-5.460938	0.408369
Se	4.567501	-4.395301	1.116437
Se	5.202230	-0.905830	-0.550288
Se	-3.298387	0.370879	4.585499
Se	-3.902509	-2.866988	2.847097
Se	-2.717682	3.963619	2.919374
Se	0.955819	4.309851	3.478400
Se	4.212654	2.889864	1.879981
Se	3.036893	5.874831	-0.274252
Se	4.688433	2.226480	-2.066066
Se	2.747418	-0.015315	-4.424751
Se	3.428214	-4.478059	-2.847555
Se	-0.011542	-3.232715	-4.095262
Se	-6.369168	-2.000981	-0.983451
Se	-3.313286	-4.305479	-0.550118
Se	-5.172417	1.561492	1.673691
Se	-4.700782	2.243268	-2.010040
Se	-1.895912	1.765875	-4.672817
Se	-3.186538	-1.723459	-4.154671
Se	-1.492790	5.279134	-0.549179
Se	0.491594	4.263998	-3.616349
P	3.185799	-0.376864	5.401861
P	0.491966	-5.641523	2.554560
P	5.887769	-2.792955	0.374269
P	-4.068610	-1.646294	4.691766
P	-1.096284	4.941770	3.987845
P	4.363445	4.960315	1.243210
P	4.370338	1.438313	-4.068531
P	1.711193	-4.545212	-4.252366
P	-5.265783	-3.880655	-1.392119
P	-5.995751	2.309075	-0.237291
P	-2.933240	0.028197	-5.462361
P	-0.555068	5.804987	-2.475023
Pd	-0.145993	0.078325	0.185436
H	-5.412829	-1.671675	5.159198

H	-3.446582	-2.405780	5.719928
H	1.569414	-6.539704	2.785218
H	-0.581549	-6.384875	3.110799
H	6.749416	-2.432265	1.441957
H	6.810775	-3.416889	-0.505222
H	-1.234399	4.766909	5.391593
H	-1.203042	6.358936	3.913682
H	4.296318	5.819928	2.371383
H	5.688339	5.225709	0.804722
H	-5.256652	-4.010674	-2.806353
H	-6.149320	-4.935262	-1.040409
H	-6.398239	3.655214	-0.031559
H	-7.256439	1.709224	-0.505544
H	2.276157	-4.456826	-5.553191
H	1.302474	-5.905995	-4.259885
H	5.590740	0.883289	-4.537255
H	4.183823	2.483307	-5.011392
H	0.268717	6.950458	-2.304699
H	-1.564641	6.337538	-3.318859
H	3.832751	-1.283342	6.287824
H	3.635220	0.857004	5.943900
H	-4.196107	0.388873	-6.009340
H	-2.294668	-0.457834	-6.635450
H	-0.430658	0.344423	1.858603

82

PdHAg20 C3a

Pd	0.000093	-0.000115	0.055292
Ag	1.046153	-1.398118	-2.237169
Ag	2.820879	0.294624	-0.594926
Ag	0.684716	1.597698	-2.243220
Ag	-1.728364	-0.213082	-2.240618
Ag	-1.154115	-2.592214	-0.590744
Ag	1.641722	-2.298295	0.457506
Ag	1.936074	0.214150	2.208674
Ag	1.169701	2.572025	0.447836
Ag	-1.666536	2.293647	-0.600478
Ag	-2.811982	-0.271880	0.451727

Ag	-0.787422	-1.778057	2.211146
Ag	-1.151202	1.575883	2.204579
Ag	-0.006526	4.608471	-1.657770
Ag	-0.687226	4.605231	1.736591
Ag	3.997932	-2.302773	-1.643687
Ag	4.328830	-1.702018	1.749442
Ag	-3.989879	-2.314493	-1.646458
Ag	-3.642867	-2.892482	1.750122
Ag	-0.001248	0.009030	4.648881
Ag	0.001512	-0.009884	-4.801702
H	0.000031	0.003183	1.776863
Se	1.631964	-2.105840	5.024743
Se	4.244299	0.282737	3.607768
Se	-2.362162	3.545230	3.599537
Se	1.014305	2.481728	5.015984
Se	-2.650164	-0.346729	5.020073
Se	-1.885399	-3.807199	3.613854
Se	2.899404	-3.972752	2.299702
Se	2.666445	-4.634780	-1.421857
Se	5.122867	1.674718	-0.702812
Se	6.232169	-1.987826	-0.110374
Se	1.994597	4.503288	2.283266
Se	2.679200	4.621478	-1.439689
Se	-1.393737	6.390898	-0.127710
Se	-4.012500	3.597589	-0.706281
Se	-5.343757	0.005560	-1.432975
Se	-4.894622	-0.517118	2.290953
Se	-4.838266	-4.402053	-0.108698
Se	-1.111002	-5.276013	-0.690898
Se	-1.432357	3.854052	-3.826089
Se	1.764551	2.029000	-4.746862
Se	-2.627168	-3.179227	-3.813921
Se	-2.645444	0.499223	-4.745094
Se	0.884653	-2.556032	-4.737941
Se	4.061726	-0.697112	-3.816670
P	3.441771	-0.928018	5.283227
P	-0.910385	3.460909	5.273580
P	-2.535443	-2.502271	5.284704

P	3.454937	-5.202763	0.540163
P	6.651111	0.138244	-0.596041
P	2.780180	5.593893	0.519414
P	-3.445374	5.689532	-0.607579
P	-6.234044	-0.387358	0.528387
P	-3.206125	-5.830173	-0.586297
P	0.274582	3.624310	-5.164194
P	-3.283350	-1.589794	-5.155536
P	3.012237	-2.065028	-5.152329
H	4.476660	-1.789152	5.729835
H	3.327840	-0.040205	6.384081
H	-3.799327	-2.966825	5.730587
H	-1.711374	-2.844411	6.387840
H	3.037537	-6.532138	0.807132
H	4.863807	-5.373772	0.485276
H	7.666227	0.540754	0.310229
H	7.388781	0.089538	-1.807543
H	4.140716	5.897020	0.783563
H	2.224573	6.899705	0.461629
H	-4.299858	6.370552	0.297881
H	-3.774009	6.348719	-1.820726
H	-7.176406	0.640236	0.790524
H	-7.087319	-1.521490	0.474954
H	-3.367259	-6.907315	0.323430
H	-3.614950	-6.448754	-1.796455
H	1.003017	4.836380	-5.284886
H	-0.207097	3.470984	-6.490306
H	-2.911972	-1.934103	-6.481291
H	-4.697443	-1.564860	-5.273993
H	3.123219	-1.576074	-6.480005
H	3.697753	-3.302445	-5.267068
H	-0.681544	4.789124	5.715721
H	-1.620940	2.921796	6.377008

82

PdHAg20 C3b

Pd	-0.000234	0.000101	-0.060434
Ag	1.611986	-0.659295	2.222067

Ag	-2.610122	1.091941	0.598199
Ag	-0.229154	1.728038	2.221055
Ag	0.360868	-2.805381	0.599844
Ag	-1.376152	-1.060060	2.225658
Ag	2.250785	1.715880	0.592546
Ag	-1.823227	0.638756	-2.236029
Ag	2.615495	-1.075388	-0.502370
Ag	0.352906	-1.900327	-2.235015
Ag	-0.377733	2.802561	-0.503859
Ag	1.463245	1.254075	-2.239729
Ag	-2.239685	-1.728588	-0.496221
Ag	0.004989	0.006165	4.790450
Ag	3.191003	-3.234645	1.559803
Ag	-4.396482	-1.141865	1.565450
Ag	1.209937	4.382797	1.552608
Ag	-3.250167	3.260908	-1.573951
Ag	-1.203350	-4.446556	-1.565938
Ag	4.448448	1.179506	-1.578199
Ag	-0.004530	-0.006246	-4.699997
H	-0.001845	-0.002158	-1.766550
Se	2.678855	-0.347096	4.753660
Se	-1.026368	2.498236	4.754033
Se	-1.637712	-2.132985	4.760352
Se	1.657075	-3.856944	3.705417
Se	5.175092	-1.419096	1.794686
Se	3.021808	-4.816123	-0.630180
Se	-4.167763	0.503407	3.706554
Se	-1.353848	5.192689	1.792688
Se	-4.520668	3.124434	0.792800
Se	4.928054	-1.445961	-1.993658
Se	-0.443611	-5.476395	0.799644
Se	-3.814160	-3.766792	1.806847
Se	2.521233	3.367996	3.696226
Se	4.966769	2.354084	0.785151
Se	-1.217346	4.988579	-1.995875
Se	-3.717072	-3.550574	-1.982057
Se	-3.949273	1.594962	-3.608106
Se	-0.510759	2.638128	-4.899058

Se	2.659307	5.024488	-0.641024
Se	-5.682241	-0.211094	-0.626582
Se	-2.042699	-1.766205	-4.891005
Se	0.583455	-4.221348	-3.604997
Se	2.538440	-0.890828	-4.896202
Se	3.354785	2.612491	-3.615966
P	2.689436	-2.538926	5.102567
P	6.090354	-1.824430	-0.141402
P	1.566771	-6.202753	0.205996
P	-4.624445	-4.362679	-0.126761
P	0.867238	3.603899	5.097319
P	4.589632	4.457931	0.190785
P	-1.466095	6.186091	-0.144085
P	-3.540962	-1.045260	5.107846
P	-6.155118	1.744866	0.203893
P	-2.664158	2.476748	-5.194099
P	-0.828092	-3.551456	-5.187128
P	3.476088	1.053576	-5.196875
H	6.544777	-3.165920	-0.229214
H	7.319163	-1.118359	-0.212955
H	4.052440	-2.906145	5.248455
H	2.199299	-2.798717	6.408616
H	2.086835	-6.871832	1.344090
H	1.412055	-7.285765	-0.697444
H	-0.061637	-3.460765	-6.377684
H	-1.694778	-4.629035	-5.503301
H	4.841740	0.841669	-5.516829
H	-6.013494	-4.085711	-0.214191
H	-4.627439	-5.780104	-0.194149
H	-6.994139	1.632498	1.342682
H	-7.016008	2.417716	-0.701199
H	-4.540222	-2.041671	5.257970
H	-3.519522	-0.486739	6.412094
H	-2.968705	1.762487	-6.381614
H	-3.164769	3.764540	-5.515483
H	-2.692400	6.896775	-0.212904
H	-0.532111	7.250597	-0.235451
H	0.503888	4.967924	5.243266

H	1.340625	3.310316	6.402375
H	4.912044	5.243675	1.327463
H	5.602772	4.864498	-0.715471
H	3.010684	1.670663	-6.386709

82

PdHAg20 C1a

Pd	0.129577	0.153090	0.127429
Ag	0.950100	1.440844	-2.325798
Ag	-1.218857	-0.547302	-2.316490
Ag	-1.631526	2.162340	-1.201006
Ag	0.926339	2.874719	0.264717
Ag	2.973546	0.668152	-0.336654
Ag	1.557990	-1.446834	-1.852530
Ag	-2.636880	-0.373548	0.226085
Ag	-1.557742	1.925908	1.732650
Ag	1.881258	0.820770	2.434406
Ag	1.997059	-1.829514	1.070096
Ag	-0.621673	-2.591836	-0.165433
Ag	-0.654802	-1.172894	2.517484
Ag	4.441735	-2.047974	-0.909260
Ag	4.697487	-0.614369	2.052744
Ag	-3.567642	-0.065190	3.131417
Ag	-2.877125	-3.065736	1.753001
Ag	-3.448612	-2.710677	-1.432741
Ag	-0.653368	-3.510415	-3.064863
Ag	0.204100	4.623337	-2.152193
Ag	-1.928683	4.761917	0.430723
H	0.038132	0.641036	1.765655
Se	1.516105	1.964843	-4.914767
Se	2.851612	4.043530	-2.052249
Se	-0.500588	6.790403	-0.664410
Se	1.561664	4.884176	1.929711
Se	-1.547149	3.622328	-3.917063
Se	-3.978146	3.734979	-1.030715
Se	-2.563866	0.098566	-4.530190
Se	-3.184713	-3.663378	-3.966475
Se	1.412071	-2.456540	-4.475974

Se	3.159335	-4.281644	-1.687861
Se	5.151481	0.124140	-2.348706
Se	5.231783	1.812572	1.040126
Se	6.131584	-2.713940	1.143867
Se	2.719979	-4.301355	1.899522
Se	3.273787	-1.071301	4.310538
Se	3.294787	2.644072	3.929672
Se	-4.707037	2.129806	2.085633
Se	-1.834606	4.314422	3.121877
Se	-1.835029	-0.269170	5.169856
Se	-1.109721	-3.684165	3.777392
Se	-5.207608	-0.644741	-0.800266
Se	-5.280792	-2.168494	2.760544
Se	-3.796419	-5.007012	0.067258
Se	-0.043266	-5.206004	-0.988944
P	3.050150	3.266734	-4.115218
P	1.259476	6.625853	0.665431
P	-3.391835	4.449039	-3.042382
P	-3.115743	-1.866299	-5.269657
P	2.898735	-3.955119	-3.839331
P	6.290127	1.184060	-0.795691
P	4.853252	-4.134297	2.259694
P	3.646691	0.918015	5.197630
P	-3.934148	3.700723	3.408520
P	-1.912141	-2.445170	5.397542
P	-6.276808	-1.488153	0.917117
P	-2.011595	-6.130857	-0.578118
H	3.279652	4.361621	-4.992356
H	4.320036	2.630811	-4.141317
H	2.397481	6.883976	-0.141828
H	1.250645	7.770891	1.502576
H	-3.385647	5.869080	-3.079118
H	-4.470913	4.169807	-3.920438
H	-2.314024	-2.237205	-6.380683
H	-4.393095	-1.797780	-5.884670
H	4.118536	-3.609089	-4.476471
H	2.618674	-5.200797	-4.460027
H	6.850935	2.335954	-1.405728

H	7.467125	0.456379	-0.475927
H	5.123324	-3.901239	3.633152
H	5.425415	-5.420633	2.083767
H	4.951332	0.964365	5.760840
H	2.860994	0.971503	6.379310
H	-4.120944	3.353054	4.771722
H	-4.805008	4.816449	3.297869
H	-1.203627	-2.781551	6.579488
H	-3.223318	-2.867253	5.742544
H	-7.086242	-2.566269	0.476661
H	-7.262656	-0.542036	1.295022
H	-1.795503	-7.145784	0.388643
H	-2.399855	-6.912419	-1.696902

82

PdHAg20 C1b

Pd	-0.004757	0.030143	0.098754
Ag	1.429329	1.828464	-1.644877
Ag	0.861743	2.469204	1.285244
Ag	-1.328966	2.453758	-0.834186
Ag	-0.848018	0.169471	-2.640626
Ag	-2.831804	-0.187959	-0.483751
Ag	-1.909483	1.321775	1.880746
Ag	1.151705	-0.488436	2.873658
Ag	2.794341	0.182732	0.467686
Ag	1.683283	-1.126889	-1.950056
Ag	-0.986125	-2.403269	-1.167251
Ag	-1.398953	-1.777260	1.736474
Ag	1.338016	-2.474600	0.671748
Ag	-1.620680	4.373712	1.591502
Ag	0.857430	4.756957	-0.747849
Ag	4.230676	0.620422	-2.205539
Ag	4.177714	-2.441552	-0.704325
Ag	3.914160	-1.945702	2.422451
Ag	-3.561554	-1.831528	-2.949330
Ag	-3.599046	-3.204479	0.151626
Ag	-4.339853	-0.704059	2.136630

H	-0.038111	0.513840	1.735833
Se	4.948396	0.568621	2.135795
Se	6.341980	-0.832539	-1.232620
Se	1.530619	-5.155505	0.569181
Se	5.132775	-4.053664	1.285523
Se	2.374160	1.251822	4.720671
Se	2.314258	-2.453565	4.553190
Se	3.098304	-0.197355	-4.510171
Se	2.903613	-3.565471	-2.823416
Se	-1.834294	-5.164411	-0.571891
Se	-1.589044	-3.368817	-3.886767
Se	-5.010259	-3.290860	2.477931
Se	-1.595499	-2.848265	4.187700
Se	-4.157680	3.460211	1.434429
Se	-3.480085	0.924779	4.140452
Se	2.788327	4.536128	1.183661
Se	0.027236	4.131111	3.708990
Se	-5.668366	-2.966286	-1.669599
Se	-5.481961	0.561128	-0.012750
Se	-3.062856	3.971901	-2.228499
Se	-1.095756	6.477274	-0.051623
Se	4.644858	3.154623	-1.397215
Se	1.514739	4.080292	-3.299606
Se	-0.732364	0.050540	-5.322953
Se	-3.945149	0.770115	-3.474698
P	6.598950	0.164853	0.712950
P	3.427480	-5.439603	1.590988
P	2.883727	-0.625651	5.664900
P	3.675563	-2.310128	-4.455545
P	-2.055034	-5.200376	-2.755351
P	-3.498258	-3.863158	3.998054
P	-4.505469	2.766150	3.487648
P	1.660019	5.352358	2.904270
P	-6.593760	-1.088437	-0.990499
P	-2.501778	6.006600	-1.702169
P	3.694549	4.224744	-3.038610
P	-2.740538	0.874723	-5.330805
H	7.242962	1.404807	0.468588

H	7.621605	-0.536718	1.401251
H	3.923525	-6.732098	1.279746
H	3.249028	-5.556732	2.994521
H	4.256503	-0.709918	6.025104
H	2.279116	-0.748663	6.944101
H	5.085726	-2.446657	-4.554001
H	3.267242	-2.888927	-5.684702
H	-1.223477	-6.245069	-3.234555
H	-3.337207	-5.698600	-3.109589
H	-3.312536	-5.259894	3.827593
H	-4.173405	-3.843005	5.246539
H	-5.905525	2.637636	3.683207
H	-4.194332	3.776328	4.434059
H	2.576378	5.592998	3.960560
H	1.216452	6.674737	2.635040
H	-7.689166	-1.443779	-0.162258
H	-7.266432	-0.516638	-2.099872
H	-3.666888	6.772938	-1.437414
H	-1.992410	6.696763	-2.833024
H	4.070423	5.589604	-2.939789
H	4.277943	3.872326	-4.282851
H	-3.498911	0.339706	-6.407451
H	-2.727777	2.249780	-5.683925

82

PdHAg20 C1c

Pd	0.176979	0.001918	0.124330
Ag	-1.209315	-0.035290	-2.371099
Ag	0.025111	-2.503996	-1.314249
Ag	-2.369902	-1.306275	0.058531
Ag	-2.093872	1.719971	-0.095870
Ag	0.442217	2.298660	-1.597970
Ag	1.730323	-0.288785	-2.343421
Ag	-0.055557	-2.346195	1.673106
Ag	-1.615025	0.268163	2.447186
Ag	0.450081	2.492646	1.420455
Ag	2.786764	1.245585	-0.054376
Ag	2.532107	-1.734580	0.091080

Ag	2.015555	-0.124712	2.499389
Ag	-2.388514	2.897005	-2.838877
Ag	-0.984964	4.616052	-0.271414
Ag	-2.411877	3.371324	2.453574
Ag	-2.918817	-2.712440	-2.498085
Ag	-1.940167	-4.365125	0.188085
Ag	-2.917217	-2.450886	2.850800
Ag	4.814499	-0.451001	-1.833202
Ag	4.978914	-0.402160	1.572497
H	0.252424	0.308310	1.794669
Se	-4.548945	2.673838	0.861078
Se	-3.237385	5.256588	-1.734452
Se	1.308320	5.032771	-1.717493
Se	-0.296728	3.019434	-4.505808
Se	-4.482989	1.197726	-2.789056
Se	-2.219388	-1.193493	-4.655754
Se	1.046335	-4.737978	-2.415027
Se	-2.727363	-5.377770	-2.243872
Se	-4.966375	-1.654093	-0.901655
Se	-4.263081	-4.496487	1.673923
Se	0.289499	-5.255684	1.468546
Se	-0.838858	-3.176793	4.406985
Se	4.371682	-3.011174	1.896995
Se	4.247661	-3.094246	-1.868875
Se	6.844095	0.342968	-0.217661
Se	4.424598	3.305169	-0.594100
Se	2.466550	-1.538364	4.816020
Se	4.308169	1.405204	3.471152
Se	1.486689	3.906220	3.426066
Se	-1.520390	5.889009	2.072255
Se	-1.973176	1.923706	4.714161
Se	-4.519948	-0.250182	3.009264
Se	3.845497	1.268957	-3.666951
Se	2.093926	-1.906734	-4.565469
P	-0.754315	-5.635219	-3.224988
P	-4.029266	0.065566	-4.598498
P	3.207786	-0.163529	-5.235606
P	-0.408114	-5.154016	3.542621

P	-5.578831	-3.433322	0.258649
P	-4.795308	4.479648	-0.388080
P	0.595120	4.887825	-3.788489
P	6.360758	2.499561	-0.033263
P	5.228429	-3.752012	-0.002002
P	-3.876754	0.829554	4.809148
P	0.405619	5.766214	3.168812
P	4.025907	-0.058761	5.106197
H	-0.936136	-5.283433	-4.587710
H	-0.571012	-7.037799	-3.339762
H	2.479069	0.600943	-6.185914
H	4.324724	-0.545790	-6.028890
H	-6.090108	-4.418268	-0.625182
H	-6.754911	-3.075281	0.965546
H	-5.967192	4.279868	-1.161098
H	-5.199123	5.557427	0.440728
H	-0.259510	5.984532	-4.077189
H	1.700644	5.176952	-4.628188
H	7.356463	3.193071	-0.768654
H	6.711125	2.856223	1.295114
H	6.629331	-3.520141	-0.048505
H	5.200953	-5.170418	0.034840
H	-3.815393	-0.044472	5.924685
H	-4.942293	1.684906	5.195118
H	0.169062	6.357995	4.437130
H	1.229410	6.756186	2.572523
H	3.805099	0.711421	6.279175
H	5.277835	-0.650636	5.427978
H	-3.963664	0.916221	-5.732040
H	-5.163631	-0.724684	-4.918273
H	0.568357	-5.754163	4.378350
H	-1.509578	-6.026015	3.750998

82

PdHAg20 C1d

Pd	-0.024698	0.013008	0.066650
Ag	-2.193016	0.565750	1.994927
Ag	0.970770	-2.687014	-0.286283

Ag	-1.841562	-2.130327	0.522436
Ag	1.047223	1.552865	2.265472
Ag	0.530450	-1.617955	2.413853
Ag	-2.746060	0.427108	-0.849818
Ag	1.853507	-0.436211	-2.066238
Ag	-0.944168	2.690522	0.246310
Ag	1.841037	2.142945	-0.565783
Ag	-0.965106	-1.464271	-2.217944
Ag	-0.415267	1.445986	-2.403373
Ag	2.737415	-0.461595	0.715848
Ag	-2.288781	-2.195260	3.604690
Ag	-1.294053	3.200731	3.302550
Ag	2.999122	-3.260505	1.944340
Ag	-3.917852	-2.226985	-1.697027
Ag	1.312392	-3.254950	-3.292302
Ag	3.971523	2.241747	1.678700
Ag	-2.950106	3.252628	-2.017441
Ag	2.295150	2.229012	-3.560184
Se	-4.020139	-0.144763	3.893041
Se	-2.875444	-4.252446	1.966189
Se	-0.000080	-2.237692	5.049125
Se	-0.886550	1.492610	5.350477
Se	-3.792238	3.404754	2.293025
Se	0.639406	4.933652	2.582105
Se	0.927247	-4.918533	2.531866
Se	-2.633744	-4.580317	-2.018799
Se	1.163916	-5.211872	-1.438769
Se	-1.865627	5.199918	-0.475561
Se	2.879727	2.304588	4.161749
Se	3.795447	-1.521935	3.860490
Se	-5.378613	-1.767146	0.534934
Se	-5.109623	1.922131	-1.068864
Se	-0.869682	-2.592983	-4.740579
Se	5.457764	0.142978	0.880326
Se	3.613581	-1.891995	-3.649325
Se	1.251449	0.531328	-5.379894
Se	-4.106919	-0.512701	-3.786933
Se	4.438605	-3.634554	-0.294443

Se	4.741463	1.766990	-2.525091
Se	3.510845	4.312415	0.009400
Se	1.065940	4.590548	-3.090650
Se	-1.911841	2.634237	-4.425736
P	-2.967886	0.890499	5.556421
P	-3.407161	5.207369	1.111499
P	2.088000	4.376420	4.117274
P	5.613931	-0.889586	2.834337
P	-4.965036	-3.688200	1.471774
P	-5.609126	0.808875	-2.915097
P	-2.158404	-4.293450	-4.125399
P	0.626996	-4.320044	4.609745
P	3.331891	-5.373580	-0.998697
P	2.997023	-0.778207	-5.464302
P	4.943946	3.717421	-1.572038
P	-0.485734	4.325504	-4.598211
H	-3.099713	6.302782	1.959483
H	-4.642670	5.631712	0.557826
H	-3.792057	1.991869	5.903984
H	-3.087739	0.083836	6.717026
H	1.589036	4.634525	5.420328
H	3.172469	5.289152	4.056381
H	4.929622	4.764552	-2.529606
H	6.264853	3.831103	-1.066373
H	-1.220389	5.534921	-4.702175
H	6.498006	-1.981998	2.637378
H	6.371162	-0.088563	3.728047
H	3.465898	-6.466704	-0.103850
H	3.994362	-5.881920	-2.145704
H	1.787389	-4.557081	5.391420
H	-0.288843	-5.225548	5.205453
H	4.145284	-0.073340	-5.909798
H	2.813541	-1.696043	-6.530858
H	-1.598206	-5.497331	-4.625459
H	-3.334524	-4.195678	-4.913422
H	-5.479800	-4.762581	0.700827
H	-5.755822	-3.794948	2.645009
H	-6.817262	0.114320	-2.647172

H	-6.037424	1.718636	-3.916453
H	0.068948	4.234161	-5.901160
H	-0.245857	0.124918	1.740032

82

PdHAg20 C3B S=1

Pd	0.030690	-0.057083	-0.035496
Ag	0.984953	2.552867	0.780706
Ag	-2.739478	-0.614498	0.746701
Ag	-1.872403	2.126029	0.005512
Ag	2.052292	0.149787	1.998784
Ag	-0.857172	0.964082	2.523146
Ag	0.302099	1.978900	-2.026526
Ag	-1.196300	-2.618449	-0.818976
Ag	2.762509	0.785375	-0.797553
Ag	2.334005	-2.006900	-0.058089
Ag	-2.050761	-0.039651	-2.067971
Ag	0.842899	-1.034406	-2.559676
Ag	-0.361794	-1.969326	1.984443
Ag	-1.246991	4.026335	2.234324
Ag	4.075151	2.308046	1.378955
Ag	-2.778008	-1.128029	3.691880
Ag	-2.496926	2.844267	-2.884125
Ag	-4.107484	-2.271502	-1.403968
Ag	2.561911	-2.725889	2.955847
Ag	2.722226	1.014742	-3.845139
Ag	1.250381	-4.055101	-2.015329
H	0.515866	-1.643905	-0.603237
Se	0.820145	5.349370	1.042374
Se	-3.682070	4.058727	0.992853
Se	-1.039329	2.792178	4.644690
Se	2.681336	3.226854	3.526126
Se	4.256594	3.800512	-0.877239
Se	5.704443	0.175954	1.818643
Se	-4.224106	1.133421	3.374969
Se	-4.846129	1.902029	-1.966418
Se	-5.552900	-0.924711	0.465294
Se	4.976131	0.431998	-2.500540

Se	3.014618	-0.545463	4.463775
Se	-0.604676	-1.016806	5.279371
Se	-1.487460	5.132989	-1.946167
Se	1.678320	3.501034	-3.899065
Se	-3.850042	-1.214424	-3.896910
Se	0.345138	-4.199277	3.399748
Se	-3.020061	-4.702314	-0.737065
Se	-0.870112	-3.919424	-3.723639
Se	-1.406528	1.562093	-4.999014
Se	-3.785840	-3.425575	2.748973
Se	1.272006	-5.706989	0.128450
Se	4.287933	-3.480275	1.035154
Se	3.682347	-3.189089	-2.969870
Se	1.458757	-0.990125	-5.167727
P	2.213385	5.221540	2.749970
P	5.606750	2.497657	-1.984956
P	5.141005	-0.141663	3.886171
P	-0.243342	-3.178666	5.284876
P	-3.150378	5.524464	-0.574214
P	0.103142	3.100617	-5.400821
P	-5.224386	0.488743	-3.599108
P	-3.185926	2.323072	4.895479
P	-5.628132	-2.531881	1.984144
P	-2.247753	-5.298069	-2.719878
P	3.381919	-5.478058	0.564836
P	3.012406	-2.582942	-4.945281
H	6.857284	2.367085	-1.326851
H	5.996228	3.173132	-3.170266
H	3.398190	5.917735	2.396040
H	1.741798	6.024991	3.820798
H	5.533401	0.944629	4.710424
H	5.938659	-1.187594	4.416825
H	4.186238	-6.013929	-0.474165
H	3.717849	-6.338824	1.640394
H	4.141564	-2.202677	-5.714418
H	-1.369261	-3.875214	5.795707
H	0.724880	-3.461253	6.283374
H	-6.421200	-2.032176	3.048918

H	-6.447144	-3.587448	1.507122
H	-3.322398	1.735032	6.179559
H	-3.908676	3.531021	5.072939
H	-1.665723	-6.585107	-2.585305
H	-3.323119	-5.580958	-3.601764
H	-6.549777	0.002012	-3.459953
H	-5.310745	1.162065	-4.844964
H	-4.340768	5.786454	-1.300063
H	-2.913601	6.788751	0.024179
H	-0.514124	4.345830	-5.686514
H	0.711032	2.806295	-6.649003
H	2.529806	-3.687616	-5.693777
